# Dual Role of a SAS10/C1D Family Protein in Ribosomal RNA Gene Expression and Processing Is Essential for Reproduction in *Arabidopsis thaliana*

**DOI:** 10.1371/journal.pgen.1006408

**Published:** 2016-10-28

**Authors:** Ying-Jiun C. Chen, Huei-Jing Wang, Guang-Yuh Jauh

**Affiliations:** 1 Institute of Plant and Microbial Biology, Academia Sinica, Taipei, Taiwan; 2 Molecular and Biological Agricultural Sciences Program, Taiwan International Graduate Program, Academia Sinica and National Chung Hsing University, Taichung, Taiwan; 3 Graduate Institute of Biotechnology, National Chung-Hsing University, Taichung, Taiwan; 4 Biotechnology Center, National Chung-Hsing University, Taichung, Taiwan; Gregor Mendel Institute of Molecular Plant Biology, AUSTRIA

## Abstract

In eukaryotic cells, ribosomal RNAs (rRNAs) are transcribed, processed, and assembled with ribosomal proteins in the nucleolus. Regulatory mechanisms of rRNA gene (rDNA) transcription and processing remain elusive in plants, especially their connection to nucleolar organization. We performed an *in silico* screen for essential genes of unknown function in *Arabidopsis thaliana* and identified *Thallo* (*THAL*) encoding a SAS10/C1D family protein. *THAL* disruption caused enlarged nucleoli in arrested embryos, aberrant processing of precursor rRNAs at the 5’ External Transcribed Spacer, and repression of the major rDNA variant (*VAR1*). *THAL* overexpression lines showed de-repression of *VAR1* and overall reversed effects on rRNA processing sites. Strikingly, *THAL* overexpression also induced formation of multiple nucleoli per nucleus phenotypic of mutants of heterochromatin factors. THAL physically associated with histone chaperone Nucleolin 1 (NUC1), histone-binding NUC2, and histone demethylase Jumonji 14 (JMJ14) in bimolecular fluorescence complementation assay, suggesting that it participates in chromatin regulation. Furthermore, investigation of truncated THAL proteins revealed that the SAS10 C-terminal domain is likely important for its function in chromatin configuration. THAL also interacted with putative Small Subunit processome components, including previously unreported Arabidopsis homologue of yeast M Phase Phosphoprotein 10 (MPP10). Our results uncovering the dual role of THAL in transcription and processing events critical for proper rRNA biogenesis and nucleolar organization during reproduction are the first to define the function of SAS10/C1D family members in plants.

## Author Summary

The rRNA regulatory network underlying the structure and function of the plant nucleolus is largely unknown. We identified a previously uncharacterized SAS10/C1D family protein THAL as a novel component important for both rRNA gene expression and processing, which further impacts nucleolar structure. *thal-2* mutant had enlarged nucleoli whereas *THAL* overexpressed cells displayed multiple nucleoli resembling heterochromatin decondensation. THAL associated with the well-known histone demethylase JMJ14, which implies not only a role of THAL in chromatin regulation but also a role of JMJ14 in rDNA transcription. In addition, THAL interacted with putative Small Subunit (SSU) processome components including nucleolin and previously unreported Arabidopsis homologue of yeast MPP10, thus our work provides initial evidence of the existence and partial constituents of the putative SSU processome in Arabidopsis. Our study adds a piece to the rRNA network puzzle, provides insight into SAS10/C1D family function and prompts investigation of THAL mammalian homologues, especially since nucleolar dysfunction is characteristic of many diseases including cancer.

## Introduction

The biogenesis of mature 5.8S, 18S, and 25S ribosomal RNAs (rRNAs) requires transcription of 45S rRNA genes (rDNA) and processing of 45S precursor rRNAs (pre-rRNAs) in the nucleolus [1]. The nucleolus is not enclosed by a membrane; its formation is driven by the active transcription of rDNA and structured by pre-rRNA processing and ribosome assembly components. rDNA units are tandemly arrayed at nucleolar organizer regions (NORs), and NORs of *Arabidopsis thaliana* (Arabidopsis) abut upon the northern telomeres of chromosomes 2 and 4 (NOR2 and NOR4, [2]). The four NORs present in a diploid cell collectively form a single nucleolus, with active rDNA decondensed inside the nucleolus where they undergo transcription and silenced rDNA in compact heterochromatin blocks at the external periphery of the nucleolus [3]. Silent rDNA units are densely methylated at their promoters and associated with modifications such as histone 3 lysine 9 methylation (H3K9me); active rDNA are hypomethylated and enriched with H3K4 trimethylation (H3K4me3) [3,4]. Currently, the rRNA regulatory network underlying the structure and function of the nucleolus remains evasive, and machinery components involved are yet to be defined.

One major component in the nucleolus is the Small Subunit (SSU) processome, a ribonucleoprotein (RNP) complex required for biogenesis of 18S rRNA and subsequent assembly and maturation of the ribosome SSU in yeast *Saccharomyces cerevisiae* [5]. It contains the U3 small nucleolar RNA (snoRNA) and U Three Proteins (UTPs), with a total of as many as 72 non-ribosomal proteins which compose numerous subcomplexes [6]. A subset of SSU processome components, called t-UTPs for their requirement for transcription, are necessary for optimal rDNA transcription and closely associated with ribosomal chromatin [7]. Therefore, rDNA transcription and pre-rRNA processing are functionally connected, but to date there are limited reports investigating the coupling and co-regulation of these two processes [8]. Although extensively studied in yeast, the SSU processome is not validated in many other organisms including Arabidopsis.

The Something About Silencing 10 (SAS10)/C1D family proteins contain the SAS10/C1D and/or SAS10 C-terminal domains. In yeast and mammals, members of this family were shown to participate in RNA processing, translational control, DNA repair, and gene silencing [9]. For instance, yeast rRNA Processing 47 (RRP47) is an exosome cofactor required for processing of rRNAs and snoRNAs [10]. RRP47 interacts with exosome catalytic subunit RRP6 via its SAS10/C1D domain [11]. Its mammalian homologue C1D functions as a DNA repair factor by interacting with and activating the catalytic subunit of the sensor of DNA double-strand breaks, DNA-Dependent Protein Kinase (DNA-PK, [12]). Both RRP47 and C1D binds RNA as well as DNA, and it was proposed that SAS10/C1D domain simultaneously serves as a platform for protein interactions and a nucleic acid binding site [9]. On the other hand, SAS10 C-terminal domain has not been formally investigated. Currently there are no published reports of any members of SAS10/C1D family in plants.

Here, we present the characterization of a member of plant SAS10/C1D family named Thallo (THAL). *thal-2* arrested embryos contained enlarged nucleoli likely caused by over-accumulated pre-rRNAs; *THAL* overexpression gave rise to multiple nucleoli which may be the result of ectopic transcription and dispersal of rDNA. The interacting partners of THAL include Nucleolin 1 (NUC1), Arabidopsis homologue of yeast M Phase Phosphoprotein 10 (AtMPP10), and Nucleolar Factor 1 (NOF1) of the putative SSU processome in Arabidopsis, and possibly NUC2 and H3K4me2/3 demethylase Jumonji 14 (JMJ14). Combining these findings, we propose that THAL contributes to both transcription and processing pathways of rRNA biogenesis and thereby impacts the organization of the nucleolus and reproductive development.

## Results

### Successful Embryogenesis Requires *THAL*

**Fig 1:**
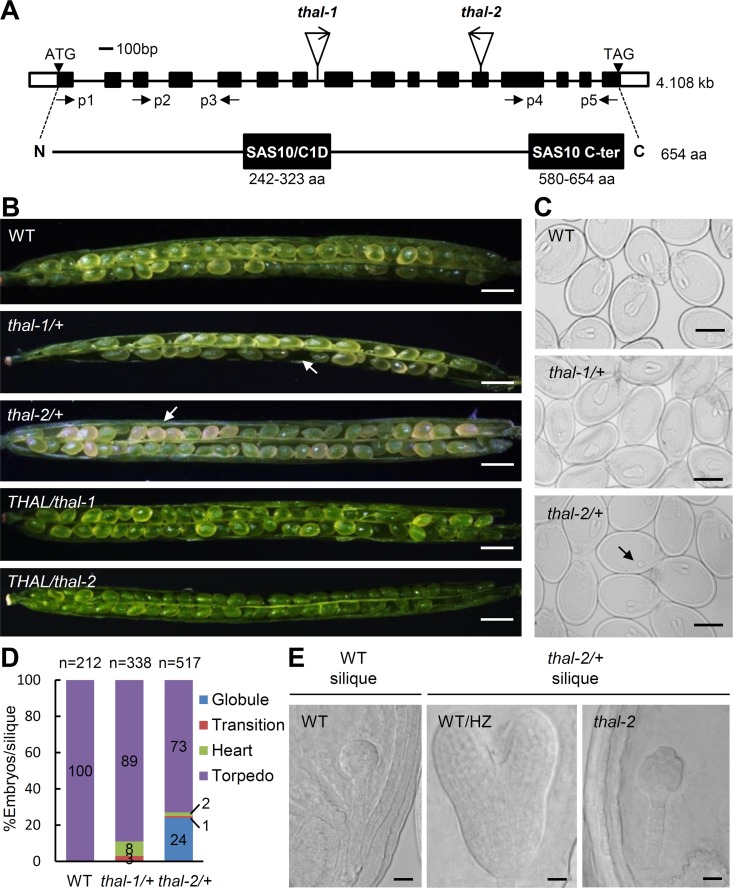
*THAL* is essential for reproductive development. (A) Schematic representation of *THAL* (*At2g43650*) genomic fragment (4.1 kilobases) and encoding protein (654 amino acids). Black and white boxes in the genomic structure indicate exons and UTRs, respectively. T-DNA insertion site of each mutant and positions of primers p1 to p5 used in S3B Fig are labelled. THAL contains two putative domains: SAS10/C1D domain (242–323 amino acid) and SAS10 C-terminal domain (580–654 amino acid), as shown in black boxes in the protein structure. (B) Seed set phenotypes of WT, *thal-1/+*, *thal-2/+*, *THAL/thal-1*, and *THAL/thal-2* siliques. Arrows indicate the undeveloped and pale-yellow seeds in *thal-1/+* and *thal-2/+* siliques, respectively. Scale bars = 0.5 mm. (C) Nomarski images of cleared seeds from WT, *thal-1/+* and *thal-2/+* siliques revealing embryonic stages. When WT/HZ embryos reached the torpedo stage in *thal-2/+* silique, a portion of embryos arrested at the globular stage (indicated by arrow). Scale bars = 250 μm. (D) Statistical analysis of embryos at the globular, transition, heart, and torpedo stages in WT, *thal-1/+* and *thal-2/+* siliques. (E) Nomarski images of cleared seeds from *thal-2/+* siliques revealing the WT/HZ torpedo and *thal-2* globular embryos. WT globular embryos were also visualized from WT siliques. Scale bars = 20 μm.

An *in silico* forward genetic screen for Arabidopsis transfer DNA (T-DNA) insertional mutants with only non-homozygous progeny from the SALK Homozygote T-DNA collection identified several mutants defective in gametophytic or embryonic development. One of these mutants harbored a T-DNA insertion in the seventh intron of *At2g43650* (Fig 1A). The encoded protein comprises two putative domains: SAS10/C1D and SAS10 C-terminal domains characteristic of SAS10/C1D family members (Fig 1A). This protein shares low (22%) identity with yeast SAS10 (S1A Fig). Phylogenetic analysis shows the widespread presence of its orthologues in other higher eukaryotes (S1B Fig), suggesting essential functions of these proteins.

The expression of *At2g43650* was detected by reverse transcription PCR (RT-PCR) in all tested tissues, including shoots, rosette and cauline leaves, flowers, siliques, roots, and seedlings, with highest expression in shoots and flowers (S2A Fig). We thus named this gene *Thallo* (*THAL*), after the Greek goddess of buds and shoots. Detailed spatial expression patterns were examined by β-glucuronidase (GUS) reporter assay, using transgenic plants expressing a GUS reporter gene under the control of a nearly 2-kb sequence upstream of *THAL* (*THALpro*::*GUS*). Significant activity of the putative *THAL* promoter was observed in the subapical region of primary roots, lateral root primordia, leaf veins, and around guard cells in seedlings, as well as the ovule, pollen, embryo, and endosperm in adult plants (S2B and S2C Fig). Collectively, *THAL* appears to be ubiquitously expressed, with preference for tissues undergoing rapid cellular growth and differentiation.

In addition to the first mutant, designated *thal-1*, another T-DNA line, *thal-2* with the T-DNA inserted in the twelfth exon was acquired (Fig 1A). Compared to wild type (WT), heterozygous (HZ) plants containing either one of these two alleles did not show any apparent morphological abnormalities during all developmental stages except in developing siliques (Fig 1B and Table 1). *thal-1/+* siliques had a portion of undeveloped seeds, resulting in an average of 37 seeds per silique, approximately three-fourths that of WT. *thal-2/+* contained one-fourth of pale-yellow seeds in siliques 10 days after pollination. These defective seed development phenotypes could be complemented by the full-length *THAL* genomic fragment (*THAL/thal*; Fig 1B and Table 1). According to the Mendelian segregation ratio, we speculated that the abortive and yellow seeds in *thal-1/+* and *thal-2/+* siliques, respectively, represented homozygous (HM) progeny. Mature dry seeds from *thal-1/+* and *thal-2/+* were plated on half MS plates and one-fourth of seeds from individual *thal-2/+* siliques failed to germinate (S2 Table). Germinated seedlings from *thal-1/+* and *thal-2/+* had a 1:1 and 2:1 ratio of HZ to WT, respectively (S1 and S2 Tables). Reciprocal crosses between *thal-1/+* and WT showed that the transmission of *thal-1* alleles is highly reduced through female gametes and slightly reduced through pollen (Table 2).

**Table 1:** Seed set phenotypes of heterozygous *thal-1/+* and *thal-2/+* and complemented *THAL/thal-1* and *THAL/thal-2*.

**Plant lines**	**Green seeds %**	**Yellow seeds %**	**Average seed number/silique**	**Total siliques counted**^a^
**WT**	100	0	52	6
***thal-1/+***	100	0	37	9
***thal-2/+***	74	26	45	12
***THAL/thal-1***	100	0	50	6
***THAL/thal-2***	100	0	51	6

^a^Siliques collected from at least five individual plants.

**Table 2:** Reciprocal cross of *thal-1/+* and WT.

Parent genotype		F1 progeny genotype			
Female	Male	HM	HZ	WT	Total
***thal-1/+***	**WT**	0	29	78	107
**WT**	***thal-1/+***	0	38	55	93

HM: homozygote; HZ: heterozygote; WT: wild type.

Developing siliques of WT, *thal-1/+* and *thal-2/+* were cleared to visualize the embryonic stages of seeds (Fig 1C). Though all seeds were presumably WT or HZ (WT/HZ) in *thal-1/+* siliques, a minority of embryos were delayed in growth (Fig 1C and 1D). In *thal-2/+* siliques, approximately one-fourth of seeds arrested uniformly at globular stage whereas the remaining three-fourths had already developed to the torpedo stage (Fig 1C and 1D). The arrested globular embryos (embryo propers) were often shaped in irregular spheres, suggesting defects in cellular division patterning (Fig 1E). In addition, they were larger than WT globular embryos because they were older (other embryos from the same silique had reached the torpedo stage).

To ascertain whether the arrested globular embryos in *thal-2/+* siliques were indeed due to disruption of *THAL*, we inspected T2 developing seeds from the *thal-2* mutant complemented by an N-terminal *GFP*-tagged *THAL* coding sequence under *THAL* native promoter (*THALpro*::*GFP-THAL/thal-2*). Clear GFP signals were detected in globular and torpedo/bent cotyledon embryos in green seeds, but not in the irregular globular embryos found in yellow seeds (S3A Fig). Given that WT embryos start to accumulate chlorophyll at the heart stage, the arrested globular embryos contributed to the pale-yellow appearance of seeds. These results strongly support the assumption that early embryo arrest is caused by loss of *THAL* function.

The T-DNA is inserted towards the C-terminal end of *THAL* in *thal-2* (Fig 1A). To determine whether *thal-2* expresses truncated *THAL*, we used total RNA extracted from green and pale-yellow immature seeds in *thal-2/+* developing siliques (hereafter bent cotyledon WT/HZ seeds and *thal-2* seeds), as well as from seeds with globular-stage embryos in WT siliques (globular WT seeds). Full-length *THAL* coding sequence was not detected by RT-PCR in *thal-2* seeds, but truncated N- and C-terminal fragments of *THAL* were both detected in low levels (S3B Fig). N-terminal fragment was produced probably because T-DNA is inserted at C-terminal end. C-terminal fragment may have been expressed by the promoter of immediate adjacent gene *At2g43660* which is in a reversed orientation downstream of *THAL*. Hence, *thal-2* is an embryo-lethal mutant that likely expresses truncated *THAL* and is used in following studies.

### THAL is a Nucleolar Protein Crucial for Nucleolar Organization

**Fig 2:**
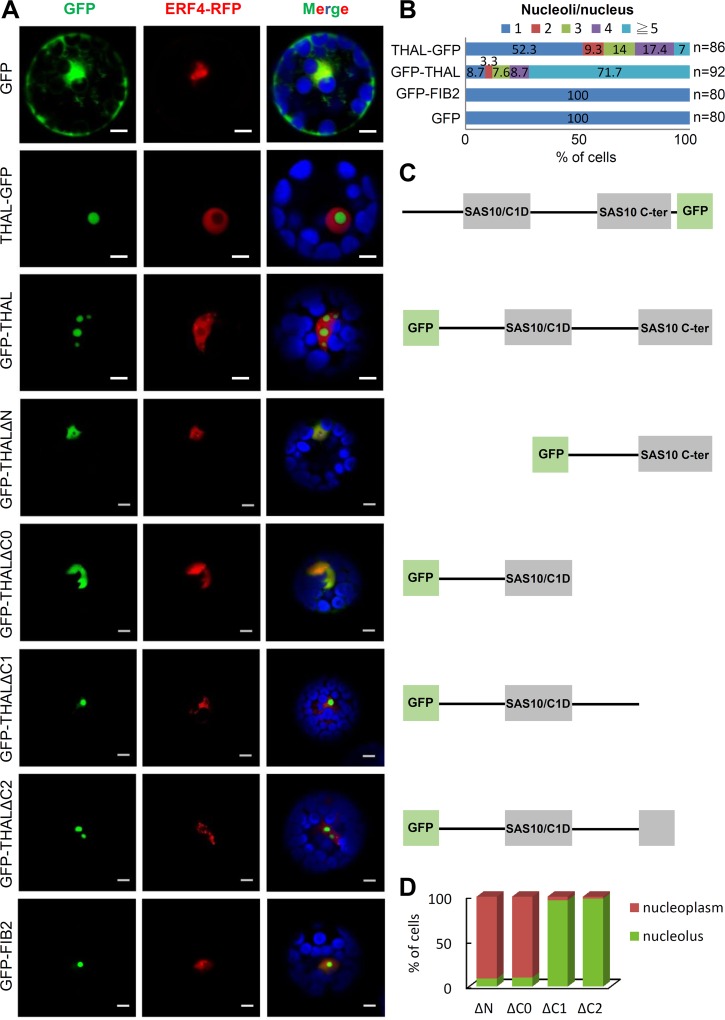
Overexpression of THAL results in multiple nucleoli. (A) Transient expression of THAL-GFP / GFP-THAL / GFP-THALΔN / GFP-THALΔC0 / GFP-THALΔC1 / GFP-THALΔC2 in protoplast cells. ERF4-RFP signals represent nuclei and blue signals represent chlorophyll autofluorescence in merged images. Scale bars = 5 μm. (B) Statistical analysis of nucleoli per nucleus when overexpressing THAL-GFP, GFP-THAL, GFP-FIB2, or GFP in protoplasts. Total number of cells counted n = 86, 92, 80, and 80, respectively. (C) Schematic representation of proteins that were overexpressed on the left in (A). (D) Statistical analysis of localization of truncated THAL proteins. Total number of cells counted n = 80 for each construct.

To explore the possible molecular function of THAL, its subcellular localization was first examined by transiently expressing a C- or N-terminal *GFP*-tagged *THAL* coding sequence (*THAL-GFP* and *GFP-THAL*) under the CaMV 35S promoter in Arabidopsis protoplasts. The control GFP alone localized in the cytoplasm and nucleus of transformed protoplasts (Fig 2A). Surprisingly, we observed recurrent multiple nucleoli in protoplast cells overexpressing THAL-GFP or GFP-THAL. The nucleolar area was defined by the absence of RFP-tagged nuclear marker Ethylene Responsive Transcription Factor 4 (ERF4-RFP), which does not localize in nucleoli. Overexpressing another nucleolar protein, Fibrillarin 2 (GFP-FIB2), resulted in only a single nucleolus. Interestingly, GFP-THAL conferred a larger effect on nucleolar dispersion than THAL-GFP, as more than 90% of GFP-THAL—but less than 50% of THAL-GFP—overexpressed protoplasts contained multiple nucleoli (Fig 2B).

Above results were validated with transgenic plants harboring *GFP-THAL* driven by *THAL* promoter (*THALpro*::*GFP-THAL*) or the estradiol-inducible XVE chimeric activator (*XVEpro*::*GFP-THAL*; S4 Fig). *THALpro*::*GFP-THAL* root cells exhibited GFP-THAL signals co-localizing with acridine orange (AO) nucleolar signals, thus confirming the nucleolar localization of THAL (S4A Fig). In *XVEpro*::*GFP-THAL* plants, 20–50% of cells displayed multiple GFP-THAL foci indicative of multiple nucleoli after estradiol treatment (S4B Fig). Taken together, the data demonstrate the nucleolar localization of THAL and that THAL overexpression induces formation of multiple nucleoli per nucleus.

The GFP-THAL fusion protein was proven functional by a complementation experiment in which GFP-THAL but not THAL-GFP gave rise to viable HM plants. This result raised the possibility that a C-terminal GFP fusion perturbed the important function of SAS10 C-terminal domain of THAL. To address this hypothesis, we investigated the domains important for THAL localization by constructing a series of truncated THAL proteins fused to GFP at the N-terminus for protoplast transient expression assay (Fig 2A, 2C and 2D). A C-terminal truncation lacking SAS10 C-terminal domain and the region between two domains (GFP-THALΔC0) resulted in co-localization with ERF4-RFP in the nucleoplasm. GFP-THALΔC1 lacking only the SAS10 C-terminal domain localized in the nucleolus but did not induce formation of multiple nucleoli. By contrast, a C-terminal truncation lacking merely half of SAS10 C-terminal domain (GFP-THALΔC2) could cause multiple nucleoli, albeit to a lesser extent than full-length GFP-THAL as most of these cells contained only two nucleoli. Finally, GFP-THALΔN with an N-terminal truncation lacking the N-terminal region and SAS10/C1D domain could no longer concentrate in the nucleolus and localized in nucleoplasm. Collectively, these results indicate that SAS10/C1D domain and the region between two domains are necessary for the nucleolar targeting of THAL, and SAS10 C-terminal domain is important for further regulation of nucleolar organization.

### THAL is Required for the Activation of Specific rDNA Variants

**Fig 3:**
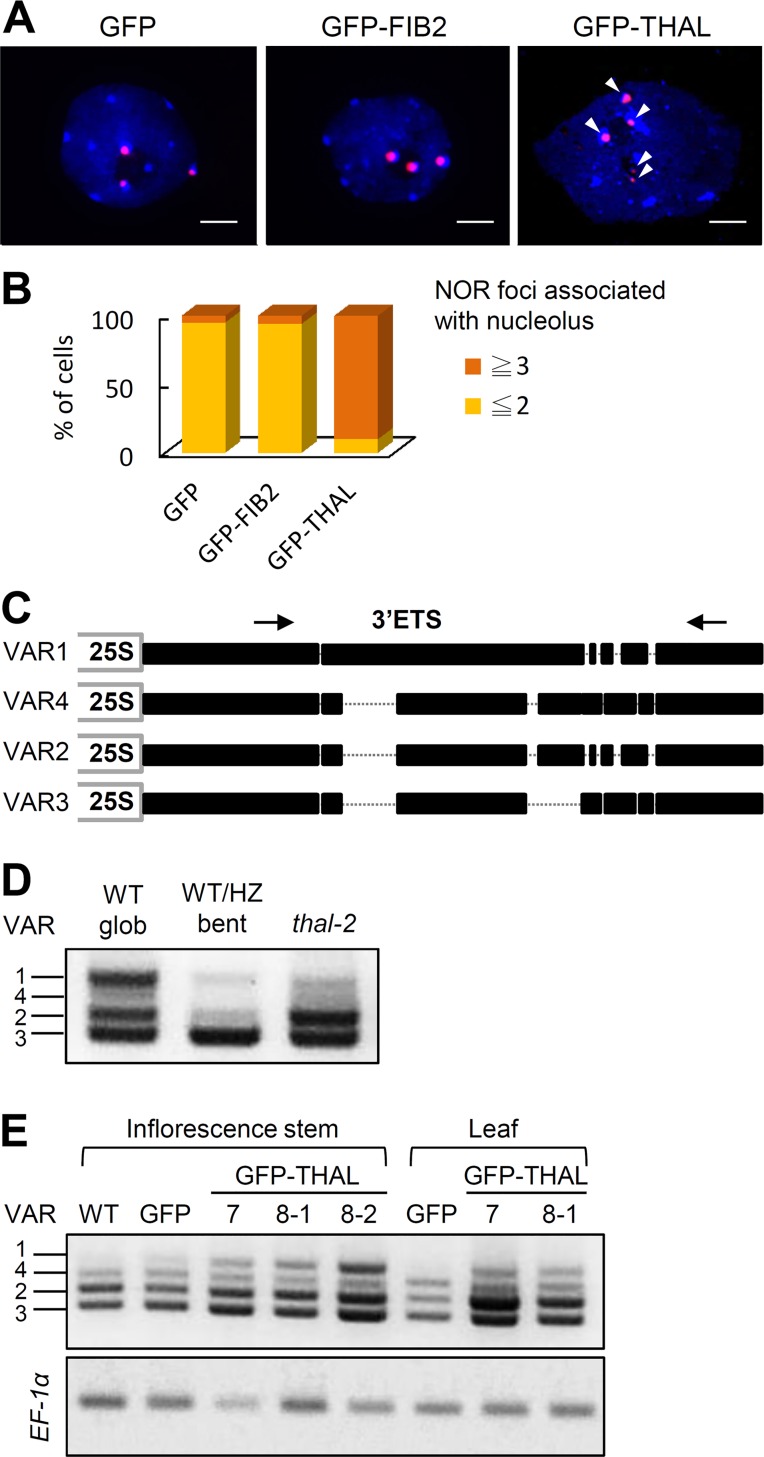
*THAL* is required for activation of rDNA *VAR1*. (A) FISH on isolated DAPI-stained nuclei (blue) of GFP, GFP-FIB2, or GFP-THAL overexpressed protoplasts with a 45S rDNA probe, revealing 45S rDNA loci (red). Arrowheads indicate 5 NOR signals observed in a GFP-THAL overexpressed nucleus. Scale bars = 2 μm. (B) Percentage of cells that contained 3 or more / 2 or less NOR foci associated with nucleolus (nucleoli) in GFP, GFP-FIB2, or GFP-THAL overexpressed protoplasts. Total cells counted n = 30 for each construct. (C) Representation of the four main rDNA variants (*VAR1*-*4*) based on insertions/deletions in the 3’ external transcribed spacer (3’ETS). Position of the primer pair used to distinguish each variant is indicated by arrows. (D) RT-PCR of globular WT (WT glob), bent cotyledon WT/HZ (WT/HZ bent), and *thal-2* seeds using a primer pair shown in (B) for the detection of relative expression levels of rDNA variants in each sample. (E) RT-PCR of WT, *35S*::*GFP*, and *35S*::*GFP-THAL* primary inflorescence stems and leaves using a primer pair shown in (B). Two individual lines of *35S*::*GFP-THAL* were examined (#7 and #8). *EF-1α* was used as an internal loading control.

It has been shown that translocated rDNA loci can retain their transcriptional activity and are able to self-assemble additional nucleoli [13]. We examined if the additional nucleoli observed in GFP-THAL (and THAL-GFP) overexpressed protoplasts are associated with NORs by fluorescence *in situ* hybridization (FISH) using 45S rDNA probes. The nucleolus contains mostly RNA and is not stained by the DNA-binding dye 4',6-diamidino-2- phenylindole (DAPI). In Arabidopsis WT interphase cells, the 4 NORs tend to coalesce so usually 3 NOR signals are detected, 2 of which are associated with the nucleolus [14]. This is the case in GFP or GFP-FIB2 overexpressed cells (Fig 3A and 3B). However, more than 4 NOR signals were discovered in protoplasts overexpressing GFP-THAL and they were mostly associated with nucleoli.

There are four main rDNA variants (*VAR1*-*VAR4*) in Arabidopsis Col-0 ecotype, based on insertions/deletions in the 3’ External Transcribed Spacer (3’ETS, [15]). Expression of each variant is differentially regulated among developmental stages and tissues. Given that multiple nucleoli observed upon THAL overexpression implies rDNA dispersal and transcription, the expression of individual rDNA variants were inspected by RT-PCR. The four variants were distinguished by a primer pair flanking the 3’ETS variable region (Fig 3C). First we analyzed THAL loss-of-function effects, using total RNA extracted from globular WT seeds, bent cotyledon WT/HZ seeds and *thal-2* seeds. Globular WT seeds showed similar expression levels of *VAR1*, *VAR2*, and *VAR3* (Fig 3D). Bent cotyledon WT/HZ seeds had highest expression of *VAR3*. *thal-2* seeds had similarly high levels of *VAR2* and *VAR3*. Therefore *VAR1*, which represents nearly 50% of rDNA, was expressed in globular WT seeds but was inhibited in globular *thal-2* seeds, suggesting that *THAL* is required for the activation of *VAR1*.

THAL gain-of-function effects were next analyzed using transgenic plants harboring CaMV 35S promoter driven *GFP-THAL* (*35S*::*GFP-THAL*). Interestingly, *35S*::*GFP-THAL* plants did not exhibit obvious morphological defects before flowering but inflorescence stems failed to elongate in 7 out of 9 individual lines (S5A Fig). Quantitative RT-PCR (qRT-PCR) analysis confirmed the overexpression of *THAL* in each line (S5B Fig). It is noteworthy that *THAL* expression was highest in WT shoots (S2A Fig). Hence, ectopic expression of *THAL* has adverse effects to plant development. We examined the expression of rDNA variants and found *VAR1* is silenced in WT and *35S*::*GFP* vegetative tissues, but is de-repressed in *35S*::*GFP-THAL* plants (Fig 3E). This is consistent with our previous results, which showed repressed *VAR1* in *thal-2* seeds. *VAR2* and *VAR3* expression also elevated in *35S*::*GFP-THAL* as compared with *35S*::*GFP* plants. We further examined the relative abundance of rDNA variants by PCR with genomic DNA and did not detect apparent differences in the proportions of rDNA variants among *35S*::*GFP-THAL*, *35S*::*GFP*, and WT (S5C Fig). Thus, THAL is required for the activation of specific variants.

### THAL Contributes to Pre-rRNA Processing at the 5’ External Transcribed Spacer

**Fig 4:**
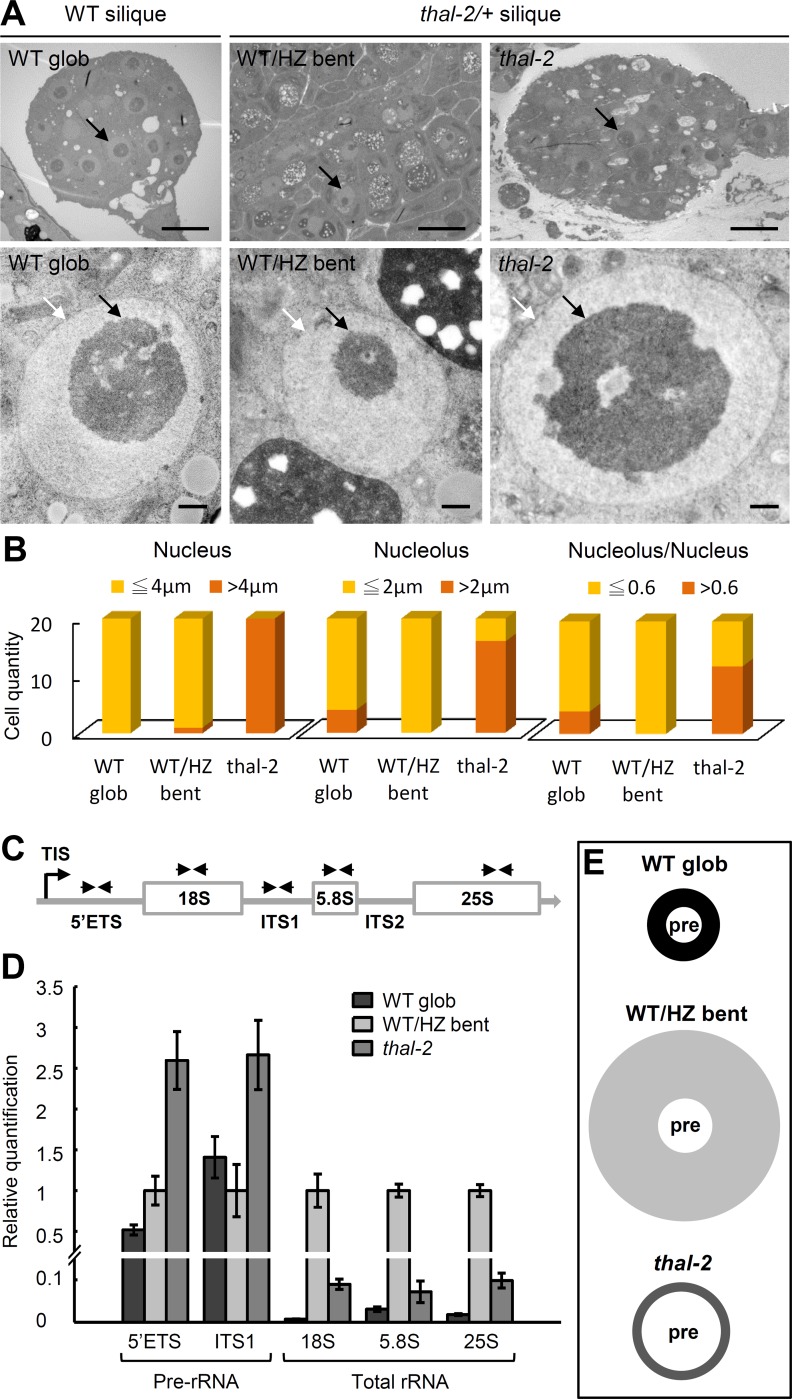
*thal-2* is defective in pre-rRNA processing. (A-B) Enlargement of nucleoli in *thal-2* embryos. (A) TEM images of WT globular (WT glob), WT/HZ bent cotyledon (WT/HZ bent), and *thal-2* embryos showing nucleoli and nuclei. White and black arrows indicate the nucleus (light-gray circle) and nucleolus (dark-gray circle), respectively. Scale bars = 10 μm (top panel) and 0.5 μm (bottom panel). (B) Measurements of nuclear and nucleolar diameters and nucleolus/nucleus ratios in WT glob, WT/HZ bent, and *thal-2* embryos. (C-E) Over-accumulation of pre-rRNA transcripts in *thal-2* seeds. (C) Representation of 45S pre-rRNA indicating positions of primer pairs used to amplify fragments containing the 5’ETS, ITS1, 18S, 5.8S, or 25S regions. TIS: transcription initiation site; ETS: external transcribed spacer; ITS: internal transcribed spacer. (D) Quantitative RT-PCR of rRNA fragments (normalized to *EF-1α*) in WT glob, WT/HZ bent, and *thal-2* seeds. Data are represented as means ± SD (n = 10). (E) Schematic diagram showing the relative proportions of pre-rRNAs (pre), mature rRNAs (shaded area), and total rRNAs (including pre-rRNAs and mature rRNAs) in WT glob, WT/HZ bent, and *thal-2* seeds using averages of two and three fragments quantified for pre-rRNAs and total rRNAs, respectively, in (D).

Loss-of-function nucleolar phenotypes in *thal-2* embryos were examined by Transmission Electron Microscopy (TEM). TEM analysis of embryo sections showed that nucleoli, nuclei, and nucleolus to nucleus ratios were mostly larger in *thal-2* embryos than those in WT/HZ bent cotyledon embryos from the same silique and those in WT globular embryos (Fig 4A and 4B). Subnucleolar structures including fibrillar center, dense fibrillar component, granular component, and nucleolar vacuole did not show prominent differences in *thal-2* (S6 Fig). We generated *thal-2/+* plants harboring a nucleolar marker FIB2-GFP by manual crossing and consistently found visibly larger nucleoli in *thal-2* embryonic cells than those in WT/HZ bent cotyledon and WT globular embryos (S7 Fig).

The enlargement of nucleoli is phenotypic of pre-rRNA processing mutants in plants [16,17]. Pre-rRNA processing includes a series of cleavage events to remove the 5’ETS, 3’ETS, and internal transcribed spacers (ITS1 and ITS2) for the generation of mature rRNAs. We examined the accumulation of pre-rRNAs in total RNA from globular WT seeds, bent cotyledon WT/HZ seeds, and *thal-2* seeds. Due to the limited amount of materials, levels of pre-rRNAs were determined by qRT-PCR with two pairs of primers, one specifically amplifying a region in 5’ETS and another flanking a region in ITS1 (Fig 4C). Additionally, three sets of primers amplifying the 18S, 5.8S, and 25S regions, respectively, were used to detect total rRNAs. Globular WT seeds had only half the fragments containing 5’ETS but similar levels of ITS1-containing pre-rRNAs as bent cotyledon WT/HZ seeds (Fig 4D). However, *thal-2* seeds accumulated approximately 2.5-fold more ITS1-containing pre-rRNAs and 5-fold more 5’ETS-containing pre-rRNAs than globular WT seeds. Total rRNA levels profoundly decreased in both *thal-2* and globular WT seeds as compared with bent cotyledon WT/HZ seeds, but levels in *thal-2* seeds were higher than those in globular WT seeds, which was likely due to the over-accumulation of pre-rRNAs (Fig 4D and 4E). Thus, compared to bent cotyledon WT/HZ seeds, *thal-2* seeds had over-accumulation of pre-rRNAs but probably much less mature rRNAs, thereby resulting in less total rRNAs; compared to globular WT seeds, *thal-2* seeds had more pre-rRNAs and total rRNAs (Fig 4E). The over-accumulation of pre-rRNA transcripts may have caused nucleoli in *thal-2* embryos to become larger than those in WT globular embryos (Fig 4A and 4B).

**Fig 5:**
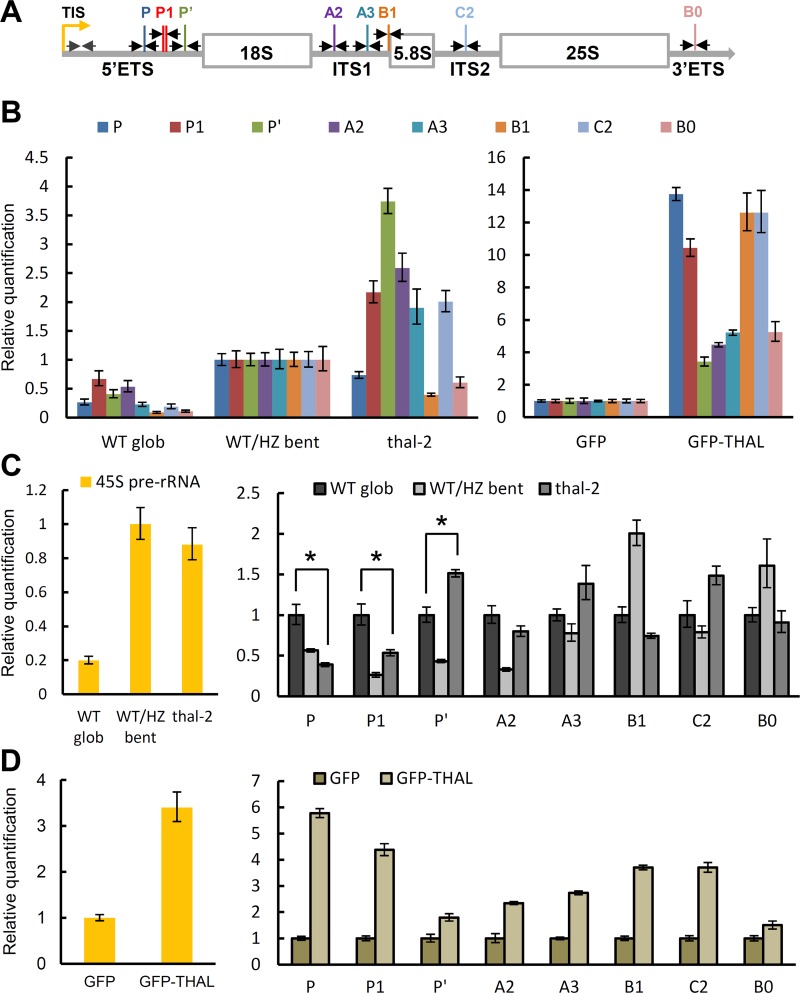
*THAL* is required for proper pre-rRNA processing at the 5’ETS. (A) Representation of the 45S pre-rRNA indicating cleavage sites relevant to this study and primer pairs (black arrows) used to detect cleavage at each site. A primer pair used to detect the full-length 45S precursor is shown in gray arrows. TIS: transcription initiation site; ETS: external transcribed spacer; ITS: internal transcribed spacer. (B) Quantitative RT-PCR of processing sites (normalized to *EF-1α*) in WT glob, WT/HZ bent, and *thal-2* seeds (left), and in *35S*:*GFP* and *35S*::*GFP-THAL* primary inflorescence stems (right). *thal-2* seeds showed a similar pattern as WT glob seeds but with higher levels. *35S*::*GFP-THAL* showed an overall opposite pattern as *thal-2*. (C) Quantitative RT-PCR of 45S pre-rRNAs (normalized to *EF-1α*, left) and processing sites (normalized to 45S precursors, right) in WT glob, WT/HZ bent, and *thal-2* seeds. Significant differences compared with WT glob seeds are indicated by * (*P* < 0.05, Student's *t* test). (D) Quantitative RT-PCR of 45S pre-rRNAs (normalized to *EF-1α*, left) and processing sites (normalized to 45S precursors, right) in *35S*:*GFP* and *35S*::*GFP-THAL* primary inflorescence stems. (B-D) Data are represented as means ± SD (n = 10 for developing seeds; n = 6 for primary inflorescence stems).

Next, various processing sites (cleavage sites) were amplified in qRT-PCR, including P, P1, P’ sites in 5’ETS, A2, A3, B1 sites in ITS1, C2 site in ITS2, and B0 site in 3’ETS. *thal-2* seeds had a similar pattern but higher levels of all cleavage sites tested as compared with globular WT seeds when *Elongation Factor 1α* (*EF-1α*) was used as an internal control (Fig 5A and 5B left panel), confirming the over-accumulation of pre-rRNA transcripts in *thal-2* seeds. However, pre-rRNA over-accumulation could result from elevated transcription and/or impaired processing. In order to exclude transcriptional accumulation and assess only processing of pre-rRNAs, we also normalized to the full-length 45S precursor determined by a primer pair immediately after the transcription initiation site (TIS; Fig 5A). Levels of nascent 45S precursors in *thal-2* seeds were 4-fold higher than in globular WT seeds and similar to bent cotyledon WT/HZ seeds (Fig 5C left panel). We found distinct accumulation patterns of cleavage sites between globular WT and bent cotyledon WT/HZ seeds, indicating that processing efficiencies differ among cleavage sites as well as embryonic stages (Fig 5C right panel). In addition, P’ site amplification level was higher while P and P1 sites were lower in *thal-2* seeds than in globular WT seeds. This suggests that THAL affects processing at the 5’ETS, of which it promotes P’ site cleavage but attenuates cleavage at P and P1 sites.

Pre-rRNA processing in *35S*::*GFP-THAL* primary inflorescence stems was next analyzed. Amplification of cleavage sites revealed a pattern overall opposite to that of *thal-2* seeds, with P’ site amplification level becoming the lowest and P site level among the highest (Fig 5B right panel). *35S*::*GFP-THAL* accumulated more than 3-fold higher levels of 45S precursors than *35S*::*GFP* (Fig 5D left panel), which may be caused by *VAR1* re-activation in *35S*::*GFP-THAL*. There was over-accumulation of all cleavage sites compared with *35S*::*GFP* even after normalization to 45S precursors (Fig 5D right panel), likely because the increased amount of 45S precursors have overwhelmed processing components. Altogether, transcription was increased and processing events were delayed in *35S*::*GFP-THAL* plants. While it is not clear how transcriptional enhancement would affect plant growth, delayed processing would further impair ribosome assembly and protein translation required for development.

Northern blots were performed with *35S*::*GFP-THAL* plants. Hybridization with S1 probe located in 5’ETS and downstream of P’ site detected the 35S (P-B0) and P-A3 precursors in *35S*::*GFP* as observed in WT of previous studies (S10 Fig, [18, 19, 20]). However fragments larger than 35S and P-A3 were observed in *35S*::*GFP-THAL*, suggesting that consistent with our qRT-PCR data, P site cleavage is indeed attenuated. S0 probe was used to further monitor the presence of pre-rRNAs containing the fragment upstream of P site, and results confirmed them over-accumulated in *35S*::*GFP-THAL*. Finally, S2 probe situated in ITS1 upstream of A3 site detected the fragments recognized by S1 probe in addition to 32S and 18S-A3 fragments. In conclusion, these results confirmed that THAL is required for processing at the 5’ETS.

### THAL Interacts with Nucleolin and AtMPP10 of the Putative SSU Processome

In an effort to identify potential candidates for the interacting partners of THAL, we performed immunoprecipitation followed by mass spectrometry (IP-MS) analysis using total proteins extracted from 8-d-old rescued *THALpro*::*GFP- THAL/thal-2* and WT seedlings. After eliminating those obtained in the WT sample, other than THAL itself, we identified several proteins involved in ribosome biogenesis: Ribosomal protein S6e (RPS6B/EMB3010), DEAD box RNA helicase RH3 (EMB1138), and Nucleolin 1 (NUC1) in the GFP-THAL immunoprecipitate (S11A Fig). Intriguingly, histone 2A proteins (H2A.1, H2A.2, H2A.X.3, H2A.X.5, H2A.W.6, H2A.W.7, H2A.Z.8, H2A.Z.9, H2A.10, H2A.Z.11, and H2A.13) were also detected. H2A.W.12 was not detected probably because of its low abundance (and *HTA4* is a pseudogene).

**Fig 6:**
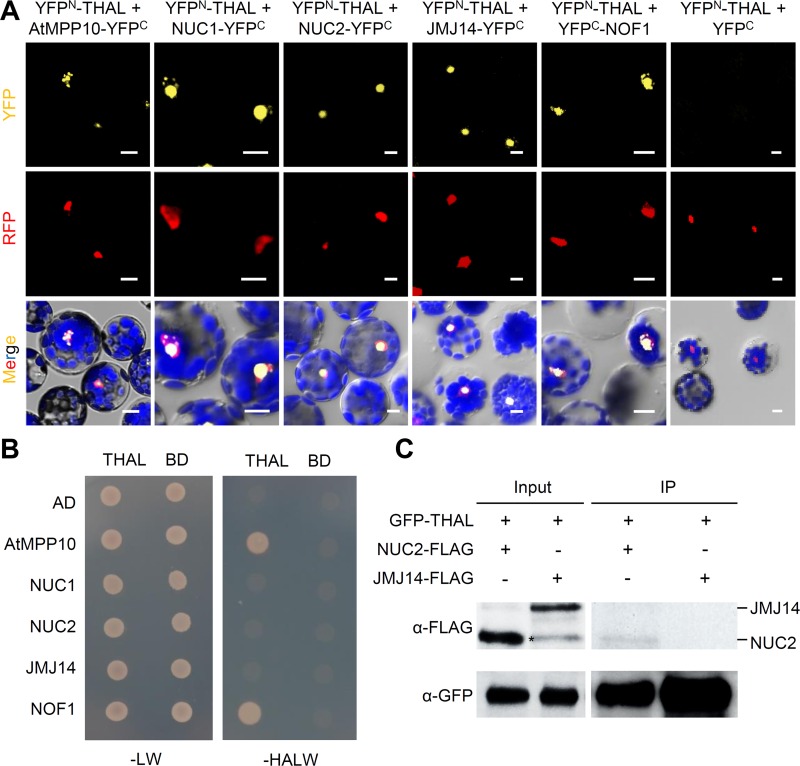
THAL interacts with nucleolin and other putative SSU components. (A) BiFC assay of YFP^N^-THAL with AtMPP10-YFP^C^, NUC1-YFP^C^, NUC2-YFP^C^, JMJ14-YFP^C^, YFP^C^-NOF1, or YFP^C^ in Arabidopsis protoplasts. RFP fused to a nuclear localization signal was co-transformed as a control for transformation efficiency and nuclear marker. Scale bars = 10μm. (B) Y2H assay of THAL with AtMPP10, NUC1, NUC2, JMJ14, or NOF1 on Leu/Trp (-LW) and His/Ade/Leu/Trp (-HALW) dropout medium. (C) Co-IP of GFP-THAL with NUC2-FLAG or JMJ14-FLAG using transient expression in seedlings. A non-specific band is marked by *.

Among the associated proteins isolated by IP-MS, of particular interest was NUC1, a multifunctional protein required for the expression of rDNA variants and NOR condensation [15]. *NUC1* was also among the genes co-expressed with *THAL* (S11C Fig). Additionally, the second nucleolin in Arabidopsis, NUC2, and three proteins selected based on protein interaction prediction by the Bio-Analytic Resource database: Arabidopsis homologue of yeast M Phase Phosphoprotein 10 (AtMPP10), Jumonji 14 (JMJ14), and Nucleolar Factor 1 (NOF1) were tested for interactions with THAL (S11B Fig). Bimolecular fluorescence complementation assay (BiFC) was conducted using Arabidopsis protoplasts transformed with THAL and candidate protein fused to the N- and C-terminal fragments of YFP, respectively (YFP^N^-THAL and NUC1-YFP^C^ etc). Protoplasts co-expressing YFP^N^-THAL and NUC1-YFP^C^ / NUC2-YFP^C^ / AtMPP10-YFP^C^ / JMJ14-YFP^C^ / YFP^C^-NOF1 all yielded clear YFP fluorescence signals in nucleoli, while negative controls YFP^N^-THAL and YFP^C^ empty vector or YFP^N^-NOT1 (unrelated protein) and NUC1-YFP^C^ / NUC2-YFP^C^ / AtMPP10-YFP^C^ / JMJ14-YFP^C^ / YFP^C^-NOF1 did not (Fig 6A and S12 Fig). Co-localization of YFP^N^-THAL and NUC1-YFP^C^ / NUC2-YFP^C^ / AtMPP10-YFP^C^ / YFP^C^-NOF1 (all nucleolar proteins and used as nucleolar markers) again demonstrates that the multiple foci are indeed nucleoli.

We further verified THAL interactions by yeast two-hybrid (Y2H) assay, in which THAL consistently interacted with AtMPP10 and NOF1 (Fig 6B). Interactions between THAL and NUC1 / NUC2 / JMJ14 were not detected, suggesting that their physical associations require a plant-specific component. Taken together, THAL associated with AtMPP10, NOF1, and NUC1 in multiple techniques (BiFC and Y2H / IP-MS), but with NUC2 and JMJ14 only in BiFC.

To further assay THAL interactions with NUC2 and JMJ14, co-immunoprecipitation (co-IP) was performed using transient expression of GFP-THAL, NUC2-FLAG, and JMJ14-FLAG in Arabidopsis seedlings. NUC2-FLAG, but not JMJ14-FLAG, could be detected in the GFP-THAL immunoprecipitates (Fig 6C). THAL and JMJ14 interaction could be specific to certain periods and conditions (e.g. developmental stage and growth status) or too transient to be detected. Likewise with NUC2 which merely showed a weak association. Nevertheless, THAL interaction with JMJ14 and NUC2 need to be further validated with caution.

## Discussion

Our results provide new insight into the regulation of rRNA biogenesis and the underlying mechanisms controlling nucleolar architecture. Studies focused on SSU processome components are still lacking in Arabidopsis, as are reports on SAS10/C1D family. Our investigation of THAL and its interacting partners provides initial evidence of the existence and partial components of the putative Arabidopsis SSU processome. Moreover, we now have a further understanding of SAS10/C1D family and the previously uncharacterized SAS10 C-terminal domain. The localization of truncated THAL proteins (GFP-THALΔC1 and GFP-THALΔC2 in Fig 2A) revealed that SAS10 C-terminal domain, of which deletion abolished the multiple nucleoli phenotype, may be important for chromatin configuration that modulates the nucleolar structure as well as rDNA transcription (see next section).

THAL shares low identity with SAS10 in yeast (S1A Fig). SAS10 was originally identified in a screen for genes that disrupted silencing upon overexpression [21]. It was later found to be a component of the SSU processome, thus is also referred to as UTP3 [5]. The homologues of SAS10 have not been functionally characterized in multi-cellular organisms except for mice [22], and due to the lethality of the mutants, genetic disruption studies have been lacking in all organisms. Indeed, both *thal-1* and *thal-2* are lethal early in reproductive development. *thal-2* development proceeded further than *thal-1* because it produced truncated *THAL* fragments, and this allowed us to perform functional experiments on *thal-2* homozygous mutants. Although the seed coat is maternal, we still detected significant differences between *thal-2* and globular WT or bent cotyledon WT/HZ seeds. We found *thal-2* seeds had repressed rDNA *VAR1*, but conversely *35*::*GFP-THAL* overexpression lines had de-repressed *VAR1* accompanied by increased expression of *VAR2* and *VAR3*. Furthermore, we observed ectopic rDNA signals associated with multiple nucleoli in a single protoplast cell overexpressing GFP-THAL (Fig 2A). Accordingly, SAS10 overproduction represses silencing at rDNA loci and telomeres [21]. We also showed that THAL is required for proper pre-rRNA processing at the 5’ETS, and lack of THAL activity abates P’ site cleavage in *thal-2* seeds. However the positive role of THAL in P’ site cleavage negatively affects the processing of nearby P and P1 sites. Again corresponding to our results, SAS10 as a part of the SSU processome is required for biogenesis of 18S but not 25S rRNA [5]. It was recently demonstrated that there are two alternative pathways for pre-rRNA processing in Arabidopsis, and P’ site cleavage is the initial step of one pathway [20,23]. Possibly, THAL promotes this particular route of processing.

**Fig 7:**
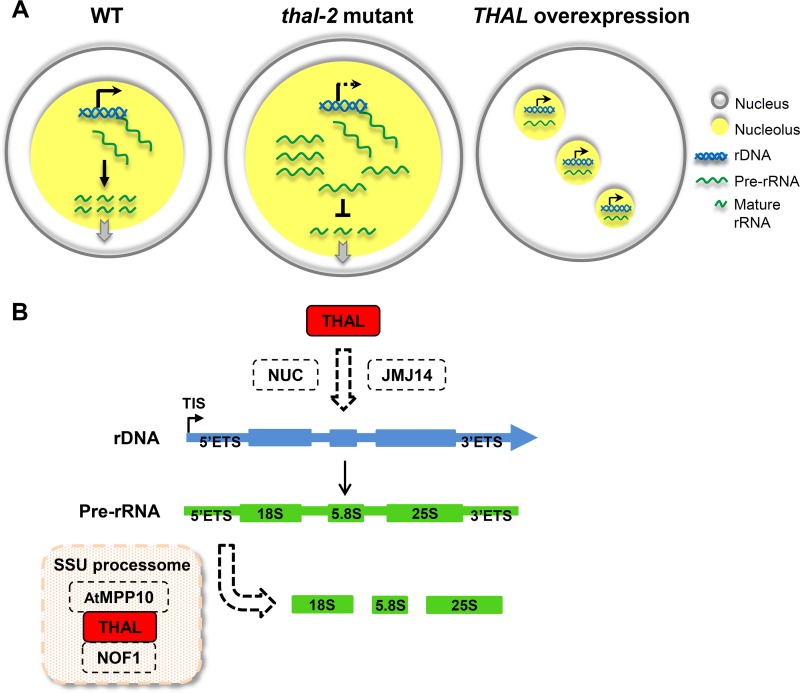
Summary of the nucleolar phenotypes and probable underlying mechanisms in *thal-2* and *THAL* overexpression plants. (A) In *thal-2* mutant, *VAR1* expression is inhibited and over-accumulation of pre-rRNA transcripts results in the enlarged nucleolus. By contrast, *THAL* overexpression re-activates *VAR1*; ectopic transcription and dispersal of rDNA elicit additional nucleoli. However, pre-rRNA processing is delayed due to excess nascent transcripts. (B) Proposed model depicting THAL interacts with NUC1 and possibly NUC2 and JMJ14 supposedly for the differential regulation of rDNA variants. Once pre-rRNA is synthesized, THAL, AtMPP10, and NOF1 but not NUC1 / NUC2 in a presumed SSU processome participate in pre-rRNA processing at the 5’ETS.

In conclusion, our study demonstrates that in *thal-2* mutant, *VAR1* expression is repressed and pre-rRNA processing is defective at the 5’ETS. Over-accumulation of pre-rRNA transcripts leads to enlarged nucleoli (Fig 7A). By contrast, overexpression of *THAL* re-activates *VAR1*. Ectopic transcription combined with dispersed rDNA elicits formation of additional nucleoli. However, pre-rRNA processing is delayed due to excess nascent transcripts from elevated transcription. The critical dual function of THAL in rRNA gene expression and processing suggests its key role as a link between these two events. SAS10 was not identified as a t-UTP subcomplex component, nor was MPP10, UTP25 (NOF1 homologue), and Nuclear Signal Recognition 1 (nucleolin homologue), though all were part of the SSU processome. Our findings of THAL and its interacting partners insinuate the presence of a novel complex implicated in this crosstalk and revealed prominent differences between the homologues from uni- and multi-cellular eukaryotes, respectively.

### THAL is a Novel Component of the rRNA Biogenesis Network and Important for Nucleolar Organization

Multiple nucleoli observed upon THAL overexpression resembles disrupted gene silencing. Previous studies have shown that interfering with essential heterochromatin regulators destabilizes nucleolar integrity [24–26]. In *Drosophila melanogaster*, H3K9 methylation and RNA interference pathways regulate the organization of rDNA and the nucleolus [24]. In Arabidopsis, telomerase-deficient cells displayed multiple nucleoli that occasionally coincided with extra rDNA signals [26], a phenomenon we repeatedly observed in GFP-THAL overexpressed protoplasts. Chromatin decondensation increases recombination between DNA repeats, which results in dispersal of rDNA and ectopic nucleoli [24]. It is thus plausible that THAL plays a negative role in chromatin condensation and gene silencing, thereby affecting nucleolar integrity that requires NOR heterochromatic structures. Accordingly, THAL associated with histone chaperone NUC1, histone-binding NUC2, and H3K4me2/3 demethylase JMJ14 in our interaction experiments.

Selective silencing of rDNA variants was just recently demonstrated to be chromosome-specific; rDNA variants located at NOR2 are silenced and those located at NOR4 are active [27]. In Col-0 vegetative tissues, *VAR1* is located at NOR2 and silenced, whereas *VAR1* introgressed into NOR4 genome is active. Therefore multiple nucleoli and de-repression of *VAR1* upon THAL overexpression are likely results of dispersal and activation of NOR2. Indeed, FISH results demonstrated more than 4 rDNA signals in GFP-THAL overexpressed protoplasts (Fig 3A and 3B). Deduced negative role of THAL in chromatin condensation would further cause this dispersal of rDNA.

There are two nucleolins found in Arabidopsis [28]. Disruption of *NUC1* causes disorganized nucleoli and NOR decondensation. In *nuc1* mutant leaves, *VAR1* is de-repressed as in *35*::*GFP-THAL* overexpression lines, thus THAL and NUC1 might play antagonistic roles in the regulation of *VAR1* expression. Alternatively, THAL and NUC1 work in concert in a complex and THAL overproduction causes a shortage of NUC1 proteins, therefore mimicking *nuc1*. A second nucleolin in Arabidopsis, NUC2, expressed only during specific developmental stages, acts antagonistically with NUC1 [14]. NUC2, along with JMJ14, AtMPP10 and NOF1, were not identified in our IP-MS experiment since we used 8-d-old seedlings in which NUC2 protein level is undetectable [14] and THAL interactions with these proteins might be stage-specific or transient. Differing from *nuc1* but similar to THAL overexpression, increased rDNA loci association with the nucleolus was observed in *nuc2*. However *VAR1* was also de-repressed in *nuc2* seedlings. Our results demonstrating THAL interacts with NUC1 and possibly NUC2 insinuate that THAL may regulate NUC1 and NUC2 functions. Further studies are anticipated to decipher the molecular mechanisms among these proteins during various developmental stages.

NUC1 binds to activated *VAR1* and conversely NUC2 binds to silent chromatin [14,15]. We hypothesize that THAL interacts with NUC1 (or NUC2, possibly depending on the developmental stage) to contribute to transcription of rDNA variants (Fig 7B). Furthermore, THAL might associate with NUC1 / NUC2 and JMJ14 at the histones to assist chromatin remodeling. However, since 45S precursors were accurately processed in *nuc1*, we deduce that THAL, AtMPP10, and NOF1 but not NUC1 / NUC2 in a presumed SSU processome participate in pre-rRNA processing at the 5’ETS.

### THAL is Essential for Reproductive Development by Tuning rRNA Biogenesis

*THAL* is primarily expressed in differentiating and dividing cells where protein synthesis is a high demand (S2B and S2C Fig). In *thal*, impaired production of mature rRNAs diminished subsequent ribosome assembly and concomitant protein translation, which terminally brought about early developmental arrest. Similar to THAL, nucleolins are multifunctional proteins implicated in various aspects of ribosome biogenesis, and *nuc1nuc2* double mutant is seedling lethal [14]. Nevertheless, pre-rRNA processing single mutants are often viable, as are gene silencing mutants [29–31]. Based on the severity of its mutant phenotypes, THAL seems to be more crucial for development than the nucleolins and a non-redundant regulator of rRNA biogenesis. It would be interesting to determine whether the dual role of THAL and its importance in nucleolar organization are evolutionarily conserved among species, especially mammals in which nucleolar enlargement is a common feature of cancer cells.

## Materials and Methods

### Plant Materials and Growth Conditions

*Arabidopsis thaliana* seeds of Col-0 ecotype were used in this study. Two T-DNA insertion lines, SALK_016916 (*thal-1*) and SALK_036872 (*thal-2*), were obtained from the Arabidopsis Biological Resource Center (ABRC). After stratification at 4°C for 48 hr, seeds were germinated in soil or half Murashige and Skoog (MS) medium solidified with 0.7% agar (pH 5.7), then grown at 22°C under a 16-hr light/8-hr dark photoperiod.

### Molecular Cloning and Generation of Transgenic Plants

The full-length genomic DNA fragment (~6.5 kb) of *THAL* and genomic fragment without the downstream putative terminator sequence (~5.5 kb) were PCR amplified using genomic DNA extracted from seedlings. The coding sequence (CDS) of *THAL* was amplified with seedling cDNA as template. For complementation of *thal* mutants, 6.5- and 5.5-kb genomic fragments were cloned into pMDC99 and pMDC107 (with GFP) vectors, respectively, and transformed into *thal/+* plants by the floral dip method. For GUS expression analysis, *THAL* native promoter fragment (*THALpro*; ~1.8 kb) was cloned into pCB308 vector and the construct was transformed into WT plants. For protoplast transient expression assay, *THAL* CDS and its truncated fragments were cloned into pGEM-T Easy vector carrying the CaMV 35S promoter and GFP sequence. To produce transgenic plants carrying *GFP-THAL*, *GFP-THAL* was isolated from the transient expression construct by treating with restriction enzymes and then cloned into pER8 (under estradiol-inducible XVE promoter; *XVEpro*) and pPZP221 vectors. *THALpro* was additionally cloned into *GFP-THAL/pPZP221*. *XVEpro*::*GFP-THAL/pER8* and *THALpro*::*GFP-THAL/pPZP221* were transformed into WT and *thal/+* plants, respectively.

### Embryo Observation

To observe embryonic stages, developing seeds in siliques from WT, *thal-1/+* and *thal-2/+* plants were cleared in a chloral hydrate solution (chloral hydrate: distilled water: glycerol 8:3:1, w/v/v) for 30 min after fixation in ethanol: acetic acid 3:1 for 1 hr. Cleared seeds were observed under an Observer Z1 microscope (Carl Zeiss). Fresh embryos were isolated from immature seeds with forceps under a microscope (Nikkon, SMZ645), then mounted in 5% glycerol for laser scanning confocal microscopy (Carl Zeiss, LSM510) to record fluorescent images.

For TEM analysis, fresh developing seeds were collected from siliques and stored in fixation buffer containing 2.5% gluteraldehyde and 4% paraformaldehyde in 0.1 M sodium phosphate buffer, pH 7.0 at 4°C before ultra-thin sectioning. Embryo sections were observed under a Philips CM 100 TEM Microscope (Philips Research) at 80 KV. Images were obtained with a Gatan Orius CCD camera.

### Protoplast Transient Expression, BiFC and FISH Assays

Protoplasts were isolated from 4-wk-old Arabidopsis WT leaves using fungal cellulase and macerozyme to remove cell walls [32]. DNA transfection was performed using the PEG-calcium solution, followed by 16-hr incubation at 24°C. As a nuclear marker, *35S*::*ERF4-RFP* was co-transformed. Empty vector and *35S*::*GFP-FIB2* were used as controls. Transformed protoplasts were observed under a laser scanning confocal microscope (Carl Zeiss, LSM510). FISH was performed as described in [33], using Arabidopsis 45S rDNA probes. Nuclei were stained with DAPI in antifade mounting medium (Vectashield, Vector Laboratories).

### Pre-rRNA Processing Analysis

Globular WT, bent cotyledon WT/HZ and *thal-2* seeds or 5-wk-old *35S*::*GFP-THAL* and *35S*::*GFP* leaves and primary inflorescence stems were collected, frozen in liquid nitrogen, and stored at -80°C before RNA extraction by the RNeasy Plant Mini Kit (Qiagen) and removal of DNA contamination by the TURBO DNA-free Kit (Applied Biosystems). Single-strand cDNA was synthesized with a random primer in order to detect rRNA transcripts. Quantitative PCR was performed using the Power SYBR Green PCR Master Mix (Applied Biosystems) with primer pairs designed by the Primer Express Software (S3 Table).

### Yeast Two-Hybrid Assay

Plasmid pairs were co-transformed into the yeast strain AH109 following the manufacturer’s instructions (Clontech). Primary transformants were first selected on Leu/Trp dropout (-LW) SD medium and confirmed again by colony PCR before growing on His/Ade/Leu/Trp dropout (-HALW) medium.

### Co-immunoprecipitation

For transient expression, Agrobacterium cultures were grown overnight and resuspended in infiltration media (5% sucrose, 5 mM MES, 200 μm acetosyringone) to 1.0 OD_600_. Arabidopsis 7-d-old AvrPto seeedlings were vacuum infiltrated (24 hr after 10 μm dexamethasone application) and collected 4 d after infiltration.

For immunoprecipitation, nuclei were first extracted with nuclei isolation buffer (0.25 M sucrose, 15 mM PIPES pH 6.8, 5 mM MgCl_2_, 60 mM KCl, 15 mM NaCl, 1 mM CaCl_2_, 0.9% Triton X-100, 1 mM PMSF) and resuspended in nuclei lysis buffer (50 mM HEPES pH 7.5, 150 mM NaCl, 1 mM EDTA, 1% Triton X-100, 0.1% DOC, 0.1% SDS, 1 mM PMSF, 1x Roche protease inhibitor cocktail) with crosslinking reagent dithiobis(succinimidyl propionate) (1 mM DSP). To quench crosslinking 50 mM Tris pH 7.5 was applied. Extracted nuclear proteins were then incubated with equilibrated GFP-trap beads (Chromotek) at 4°C for 1.5 hr under gentle agitation, followed by 3 times of washing with wash buffer (50 mM Tris pH 7.5, 150 mM NaCl). Western blots were performed using α-GFP (Santa Cruz) or α-FLAG antibodies (Sigma).

### Accession Numbers

Sequence data referred in this article can be found in the Arabidopsis Genome Initiative or GenBank/EMBL databases under the following accession numbers: AT2G43650 (*THAL*), AT1G48920 (*NUC1*), AT3G18610 (*NUC2*), AT5G66540 (*AtMPP10*), AT1G17690 (*NOF1*), AT4G20400 (*JMJ14*), AT4G25630 (*FIB2*), and AT2G37620 (*ACT1*).

## Supporting Information

S1 Fig Sequence analysis of THAL and its orthologues. Full amino-acid sequence alignment of THAL in Arabidopsis, SAS10 in *Saccharomyces cerevisiae* (Sc), and other orthologues in *Solanum demissum* (Sd), *Glycine max* (Gm), and *Mus musculus* (Mm) by *ClustalW2 multiple sequence alignment*. Identical residues in all organisms are highlighted in yellow and identical residues in THAL and other but not all organisms are highlighted in green. The SAS10/C1D and SAS10 C-terminal domains in THAL are marked by red lines.Rooted phylogenetic tree constructed by the UPGMA method representing the distances between THAL and its orthologues in various organisms including Sd, Gm, *Zea mays* (Zm), *Sorghum bicolor* (Sb), *Oryza sativa* (Os), Mm, *Homo sapiens* (Hs), *Danio rerio* (Dr), *Drosophila melanogaster* (Dm), and Sc (SAS10) after full amino-acid sequence alignment.
(TIF)

S2 Fig *THAL* is ubiquitously expressed with preference to differentiating tissues. The expression profile of *THAL* in examined tissues by RT-PCR. *ACT1* was an internal loading control to ensure equal loading of cDNA. Primer pair for *ACT1* spanned an intron and revealed no genomic DNA contamination in all cDNAs. SH, shoot; RL, rosette leaf; CL, cauline leaf; FL, flower; SQ, silique; SL, seedling; RT, root; G, genomic DNA; B, blank without DNA template. *THAL* and *ACT1* were PCR amplified with 27 and 23 cycles, respectively.(B–C) GUS staining of *THALpro*::*GUS* transgenic plants for *THAL* promoter activity in seedlings (B) and reproductive tissues (C). In seedlings (B1), GUS signals were significant in the lateral root primordia (B2), subapical region of the primary root (B3), leaf veins and around guard cells (B4 and B5). *THALpro*::*GUS* expression at the reproductive stage was observed in pollen and pollen tubes (C1-C3), ovule (C4), endosperm and embryo (C5 and C6). Scale bars = 0.2 mm (B1 and C1) and 100 μm (B2-B5, C2-C6).
(TIF)

S3 Fig *thal-2* mutant embryo lacks full-length *THAL*. Embryo development in rescued *thal-2* siliques. GFP signals in complemented globular and bent cotyledon embryos in green seeds and non-complemented globular embryos in yellow seeds (T2 seeds) from *THALpro*::*GFP-THAL /thal-2* T1 plant. Scale bars = 10 μm (globular embryos) and 100 μm (bent cotyledon embryos).RT-PCR of globular WT (WT glob), bent cotyledon WT/HZ (WT/HZ bent), and *thal-2* seeds using indicated primers (positions shown in Fig 1A). *ACT1* was an internal control. G, genomic DNA.
(TIF)

S4 Fig Nucleolar localization of THAL and subcellular phenotype of THAL overexpression in transgenic plants. Confocal microscopy of roots of 10-d-old seedlings expressing *THALpro*::*GFP-THAL* (A) and 14-d-old seedlings expressing *XVEpro*::*GFP-THAL* (B). Propidium iodide (PI) or DAPI was used to stain nuclei. Acridine orange (AO) was used to mark nucleoli and only red fluorescence was registered.THAL localized in nucleoli of *THALpro*::*GFP-THAL* plants. Scale bars = 20 μm.THAL localized in multiple nucleoli within a PI-stained nucleus of *XVEpro*::*GFP-THAL* plants upon estradiol treatment. *35S*::*FIB2-GFP* seedlings treated with estradiol did not display multiple nucleoli per nucleus. DMSO treatment did not induce GFP signals. Scale bars = 20 μm (images showing numerous cells) or 5 μm (single cell). Cells containing multiple GFP foci within a nucleus were quantified in three individual lines (Total cells n = 200, 200, 100 for #7, #8, #9, respectively).
(TIF)

S5 Fig *35S*::*GFP-THAL* plants are defective in elongation of inflorescence stems. Phenotype of *35S*::*GFP-THAL* plants, showing lines #5, 7, 8, and 9.Quantitative RT-PCR analysis of *THAL* expression in *35S*::*GFP-THAL* (lines #5, 7, 8, and 9), *35S*::*GFP*, and *XVEpro*::*GFP-THAL* upon estradiol (EST) or DMSO treatment relative to that in WT. Data are represented as means ± SD (n = 4).PCR analysis of rDNA variants using the primer pair shown in Fig 3C. *EF-1α* was an internal control.
(TIF)

S6 Fig Subnucleolar structures in *thal-2* embryo. TEM images of subnucleolar structures in WT globular, WT/HZ bent cotyledon, and *thal-2* embryos. FC, fibrillar center; DFC, dense fibrillar component; GC, granular component; NC, nucleolar vacuole. Scale bars = 200 μm.
(TIF)

S7 Fig *thal-2* embryonic cells contain enlarged nucleoli. Nucleolar size of WT torpedo, WT globular, and *thal-2* embryonic cells shown by nucleolar marker *35S*::*FIB2-GFP*. Scale bars = 20 μm. WT globular and *thal-2* embryo images are shown in the same scale.
(TIF)

S8 Fig *thal-2* over-accumulates pre-rRNAs. Quantitative RT-PCR of rRNA fragments normalized to *ACT 1* in WT glob, WT/HZ bent, and *thal-2* seeds (corresponding to Fig 4D). Data are represented as means ± SD (n = 10).
(TIF)

S9 Fig *thal-2* has abberant pre-rRNA processing. Quantitative RT-PCR of processing sites (normalized to *EF-1α*) in WT glob, WT/HZ bent, and *thal-2* seeds, using WT glob to standardize (corresponding to Fig 5B). *thal-2* seeds still showed an overall opposite amplification pattern as *35S*::*GFP-THAL* (Fig 5B). Quantitative RT-PCR of processing sites (normalized to 45S precursors) in WT glob, WT/HZ bent, and *thal-2* seeds, using WT glob to standardize (corresponding to Fig 5C).
(TIF)

S10 Fig *THAL* overexpression shows processing defect at 5’ETS. Schematic illustration of pre-rRNA processing events in Arabidopsis, showing processing site cleavages and precursors relevant to this study. Positions of S0, S1, and S2 probes used for northern blot analysis are indicated (blue, black, and purple bars, respectively). First cleavage at B0 site terminates transcription. The following 5’ splicing and P site cleavage generate 35S precursor, which can be processed by two alternative pathways to produce mature rRNAs. Northern blot analysis of processing in *35S*::*GFP-THAL*. Ethidium bromide (EtBr) staining is shown as a loading control. S0 probe detected pre-rRNAs containing the fragment upstream of P site. Using the S1 probe, 35S and P-A3 precursors were detected in *35S*::*GFP* (other intermediates are less detectable [18]). Fragments larger than 35S and P-A3 were detected in*35S*::*GFP-THAL*, suggesting attenuated cleavage at P site. S2 probe detected those recognized by S1 probe as well as 32S and 18S-A3 fragments.
(TIF)

S11 Fig Potential interacting proteins of THAL. Graph shows interesting associated proteins of THAL detected by IP-MS using 8-d-old *THALpro*::*GFP-THAL* seedlings, eliminating those detected in WT. Putative interacting proteins of THAL presented by the Arabidopsis Interaction Viewer using the Bio-Analytic Resource (BAR) database. Co-expression analysis of *THAL* by the BAR Expression Angler. *THAL* (*EMB2777*) and *NUC1* (*ATNUC-L1*) are indicated by black arrows on the right.
(TIF)

S12 Fig THAL interacts with putative SSU components and JMJ14 in BiFC. An unrelated protein YFP^N^-NOT1 and YFP^C^ were used as negative controls. RFP fused to a nuclear localization signal was co-transformed as a marker for transformation efficiency and nuclei. Scale bars = 50μm. Total of 100 cells containing YFP signals were quantified for each interaction, and only cells with RFP signals were taken into account.
(TIF)

S1 Table Segregation ratio of germinated progeny of *thal-1/+*. (PDF)

S2 Table Segregation ratio of progeny of *thal-2/+*. (PDF)

S3 Table Primers used in this study. (PDF)

S1 Text Supporting materials and methods. (PDF)

S1 Dataset Original quantification values obtained in qRT-PCR. (XLSX)

## References

[1] Tschochner H, Hurt E. Pre-ribosomes on the road from the nucleolus to the cytoplasm. Trends Cell Biol. 2003; 13: 255–263. 12742169 10.1016/s0962-8924(03)00054-0

[2] Copenhaver GP, Pikaard CS. RFLP and physical mapping with an rDNA-specific endonuclease reveals that nucleolus organizer regions of *Arabidopsis thaliana* adjoin the telomeres on chromosomes 2 and 4. Plant J. 1996; 9: 259–272. 8820610 10.1046/j.1365-313x.1996.09020259.x

[3] Pontvianne F, Blevins T, Chandrasekhara C, Mozgová I, Hassel C, Pontes OMF, et al Subnuclear partitioning of rRNA genes between the nucleolus and nucleoplasm reflects alternative epiallelic states. Genes Dev. 2013; 27: 1545–1550. 10.1101/gad.221648.113 23873938 PMC3731543

[4] Lawrence RJ, Earley K, Pontes O, Silva M, Chen ZJ, Neves N, et al A Concerted DNA Methylation/Histone Methylation Switch Regulates rRNA Gene Dosage Control and Nucleolar Dominance. Mol Cell. 2004; 13: 599–609. 14992728 10.1016/s1097-2765(04)00064-4

[5] Dragon F, Gallagher JEG, Compagnone-Post PA, Mitchell BM, Porwancher KA, Wehner KA, et al A large nucleolar U3 ribonucleoprotein required for 18S ribosomal RNA biogenesis. Nature. 2002; 417: 967–970. 10.1038/nature00769 12068309 PMC11487672

[6] Lim YH, Charette JM, Baserga SJ. Assembling a Protein-Protein Interaction Map of the SSU Processome from Existing Datasets. PLoS One. 2011; 6: e17701 10.1371/journal.pone.0017701 21423703 PMC3053386

[7] Gallagher JEG, Dunbar DA, Granneman S, Mitchell BM, Osheim Y, Beyer AL, et al RNA polymerase I transcription and pre-rRNA processing are linked by specific SSU processome components. Genes Dev. 2004; 18: 2506–2517. 10.1101/gad.1226604 15489292 PMC529538

[8] Granneman S, Baserga SJ. Crosstalk in gene expression: coupling and co-regulation of rDNA transcription, pre-ribosome assembly and pre-rRNA processing. Curr Opin Cell Biol. 2005; 17: 281–286. 10.1016/j.ceb.2005.04.001 15901498

[9] Mitchell P. Rrp47 and the function of the Sas10/C1D domain. Biochem Soc Trans. 2010; 38: 1088–1092. 10.1042/BST0381088 20659009

[10] Mitchell P, Petfalski E, Houalla R, Podtelejnikov A, Mann M, Tollervey D. Rrp47p is an Exosome-associated Protein Required for the 3’ Processing of Stable RNAs. Mol Cell Biol. 2003; 23: 6982–6992. 10.1128/MCB.23.19.6982-6992.2003 12972615 PMC193929

[11] Costello JL, Stead JA, Feigenbutz M, Jones RM, Mitchell P. The C-terminal Region of the Exosome-associated Protein Rrp47 is Specifically Required for Box C/D Small Nucleolar RNA 3’-Maturation. J Biol Chem. 2011; 286: 4535–4543. 10.1074/jbc.M110.162826 21135092 PMC3039359

[12] Yavuzer U, Smith G, Bliss T, Werner D, Jackson S. DNA end-independent activation of DNA-PK mediated via association with the DNA-binding protein C1D. Genes Dev. 1998; 12: 2188–2199. 9679063 10.1101/gad.12.14.2188 PMC317006

[13] McClintock B. The relation of a particular chromosomal element to the development of the nucleoli in *Zea mays*. Z Zellforsch Mikr Anat. 1934; 21: 283–327.

[14] Durut N, Abou-Ellail M, Pontvianne F, Das S, Kojima H, Ukai S, et al A Duplicated NUCLEOLIN Gene with Antagonistic Activity Is Required for Chromatin Organization of Silent 45S rDNA in *Arabidopsis*. Plant Cell. 2014; 26: 1330–1344. 10.1105/tpc.114.123893 24668745 PMC4001387

[15] Pontvianne F, Abou-Ellail M, Douet J, Comella P, Matia I, Chandrasekhara C, et al Nucleolin Is Required for DNA Methylation State and the Expression of rRNA Gene Variants in *Arabidopsis thaliana*. PLoS Genet. 2010; 6: e1001225 10.1371/journal.pgen.1001225 21124873 PMC2991258

[16] Lahmy S, Guilleminot J, Cheng CM, Bechtold N, Albert S, Pelletier G, et al DOMINO1, a member of a small plant-specific gene family, encodes a protein essential for nuclear and nucleolar functions. Plant J. 2004; 39: 809–820. 10.1111/j.1365-313X.2004.02166.x 15341625

[17] Abbasi N, Kim HB, Park NI, Kim HS, Kim YK, Park YI, et al APUM23, a nucleolar Puf domain protein, is involved in pre-ribosomal RNA processing and normal growth patterning in Arabidopsis. Plant J. 2010; 64: 960–976. 10.1111/j.1365-313X.2010.04393.x 21143677

[18] Weis BL, Kovacevic J, Missbach S, Schleiff E. Plant-Specific Features of Ribosome Biogenesis. Trends Plant Sci. 2015; 20: 729–740. 10.1016/j.tplants.2015.07.003 26459664

[19] Sikorski PJ, Zuber H, Philippe L, Sement FM, Canday J, Kufel J, et al Distinct 18S rRNA precursors are targets of the exosome complex, the exoribonuclease RRP6L2 and the terminal nucleotidyltransferase TRL in *Arabidopsis thaliana*. Plant J. 2015; 83: 991–1004. 10.1111/tpj.12943 26216451

[20] Hang R, Liu C, Ahmad A, Zhang Y, Lu F, Cao X. Arabidopsis protein arginine methyltransferase 3 is required for ribosome biogenesis by affecting precursor ribosomal RNA processing. Proc Natl Acad Sci. 2014; 111: 16190–16195. 10.1073/pnas.1412697111 25352672 PMC4234621

[21] Kamakaka RT, Rine J. Sir- and Silencer-Independent Disruption of Silencing in Saccharomyces by Sas10p. Genetics. 1998; 149: 903–914. 9611201 10.1093/genetics/149.2.903 PMC1460156

[22] Park SK, Lim JH, Kang CJ. Crlz1 activates transcription by mobilizing cytoplasmic CBFβ into the nucleus. BBA-Gene Regul Mech. 2009; 1789: 702–708. 10.1016/j.bbagrm.2009.08.011 19735751

[23] Weis BL, Palm D, Missbach S, Bohnsack MT, Schleiff E. atBRX1-1 and atBRX1-2 are involved in an alternative rRNA processing pathway in *Arabidopsis thaliana*. RNA. 2015; 21: 415–425. 10.1261/rna.047563.114 25605960 PMC4338337

[24] Peng JC, Karpen GH. H3K9 methylation and RNA interference regulate nucleolar organization and repeated DNA stability. Nat Cell Biol. 2007; 9: 25–35. 10.1038/ncb1514 17159999 PMC2819265

[25] Padeken J, Mendiburo MJ, Chlamydas S, Schwarz HJ, Kremmer E, Heun P. The Nucleoplasmin Homolog NLP Mediates Centromere Clustering and Anchoring to the Nucleolus. Mol Cell. 2013; 50: 236–249. 10.1016/j.molcel.2013.03.002 23562326

[26] Siroky J, Zluvova J, Riha K, Shippen DE, Vyskot B. Rearrangements of ribosomal DNA clusters in late generation telomerase-deficient *Arabidopsis*. Chromosoma. 2003; 112: 116–123. 10.1007/s00412-003-0251-7 14579127

[27] Chandrasekhara C, Mohannath G, Blevins T, Pontvianne F, Pikaard CS. Chromosome-specific NOR inactivation explains selective rRNA gene silencing and dosage control in *Arabidopsis*. Genes Dev. 2016; 30: 177–190. 10.1101/gad.273755.115 26744421 PMC4719308

[28] Pontvianne F, Matía I, Douet J, Tourmente S, Medina FJ, Echeverria M, et al Characterization of AtNUC-L1 Reveals a Central Role of Nucleolin in Nucleolus Organization and Silencing of AtNUC-L2 Gene in *Arabidopsis*. Mol Biol Cell. 2007; 18: 369–379. 10.1091/mbc.E06-08-0751 17108323 PMC1783796

[29] Lange H, Sement FM, Gagliardi D. MTR4, a putative RNA helicase and exosome co-factor, is required for proper rRNA biogenesis and development in *Arabidopsis thaliana*. Plant J. 2011; 68: 51–63. 10.1111/j.1365-313X.2011.04675.x 21682783

[30] Ohbayashi I, Konishi M, Ebine K, Sugiyama M. Genetic identification of Arabidopsis RID2 as an essential factor involved in pre-rRNA processing. Plant J. 2011; 67: 49–60. 10.1111/j.1365-313X.2011.04574.x 21401745

[31] Rodor J, Jobet E, Bizarro J, Vignols F, Carles C, Suzuki T, et al AtNUFIP, an essential protein for plant development, reveals the impact of snoRNA gene organisation on the assembly of snoRNPs and rRNA methylation in *Arabidopsis thaliana*. Plant J. 2011; 65: 807–819. 10.1111/j.1365-313X.2010.04468.x 21261762

[32] Yoo SD, Cho YH, Sheen J. Arabidopsis mesophyll protoplasts: a versatile cell system for transient gene expression analysis. Nat Protoc. 2007; 2: 1565–1572. 10.1038/nprot.2007.199 17585298

[33] Chung MC, Lee YI, Cheng YY, Chou YJ, Lu CF. Chromosomal polymorphism of ribosomal genes in the genus *Oryza*. Theor Appl Genet. 2008; 116: 745–753. 10.1007/s00122-007-0705-z 18214422 PMC2271086

