# Supplementary figures and images for: Dual Role of a SAS10/C1D Family Protein in Ribosomal RNA Gene Expression and Processing Is Essential for Reproduction in Arabidopsis thaliana
Source: PLoS Genet. 2016 Oct 28;12(10):e1006408. doi: 10.1371/journal.pgen.1006408 (PMC5085252; doi:10.1371/journal.pgen.1006408)

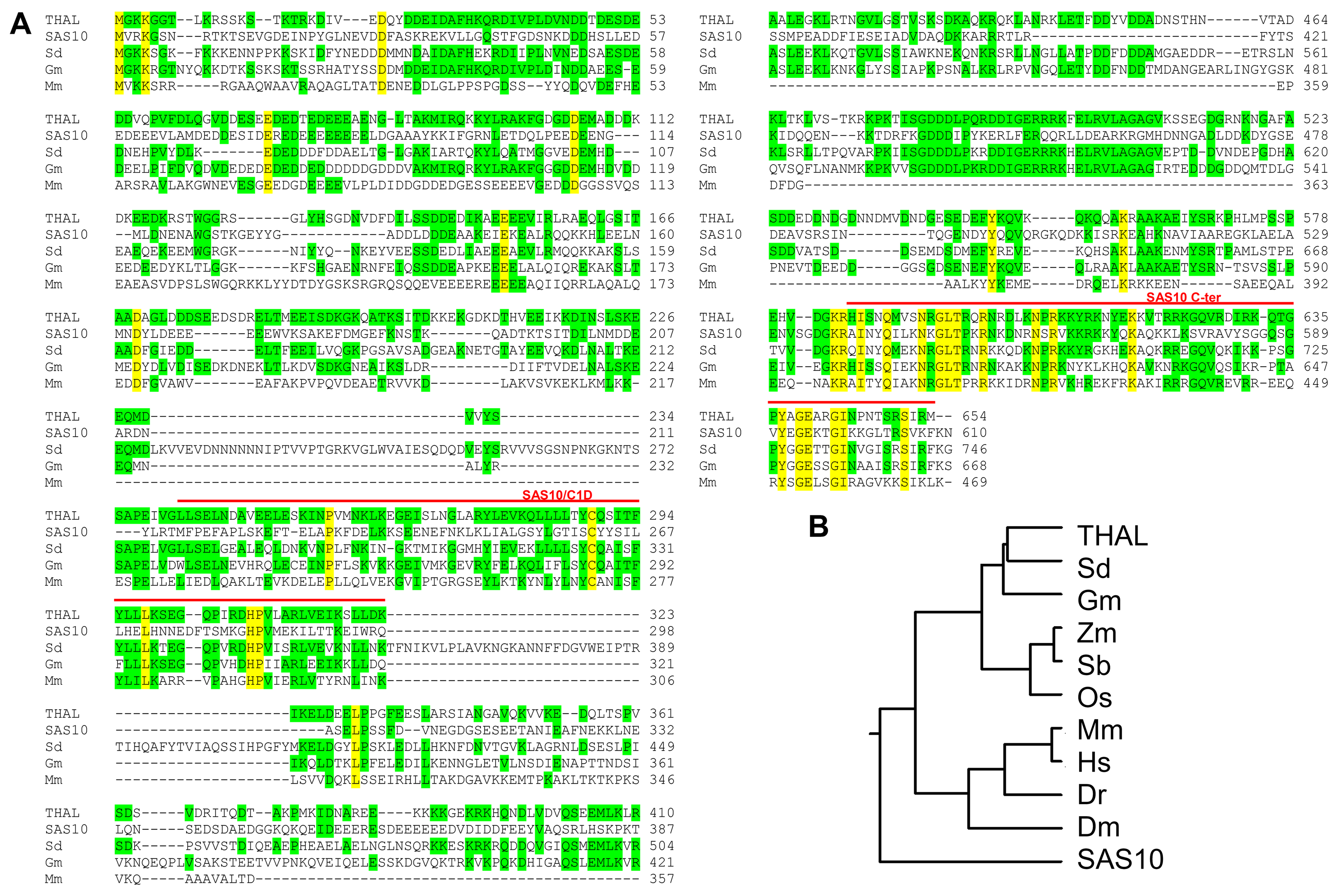

Supplement: S1 Fig — Full amino-acid sequence alignment of THAL in Arabidopsis, SAS10 in Saccharomyces cerevisiae (Sc), and other orthologues in Solanum demissum (Sd), Glycine max (Gm), and Mus musculus (Mm) by ClustalW2 multiple sequence alignment. Identical residues in all organisms are highlighted in yellow and identical residues in THAL and other but not all organisms are highlighted in green. The SAS10/C1D and SAS10 C-terminal domains in THAL are marked by red lines.Rooted phylogenetic tree constructed by the UPGMA method representing the distances between THAL and its orthologues in various organisms including Sd, Gm, Zea mays (Zm), Sorghum bicolor (Sb), Oryza sativa (Os), Mm, Homo sapiens (Hs), Danio rerio (Dr), Drosophila melanogaster (Dm), and Sc (SAS10) after full amino-acid sequence alignment. (TIF) [file pgen.1006408.s001.tif]

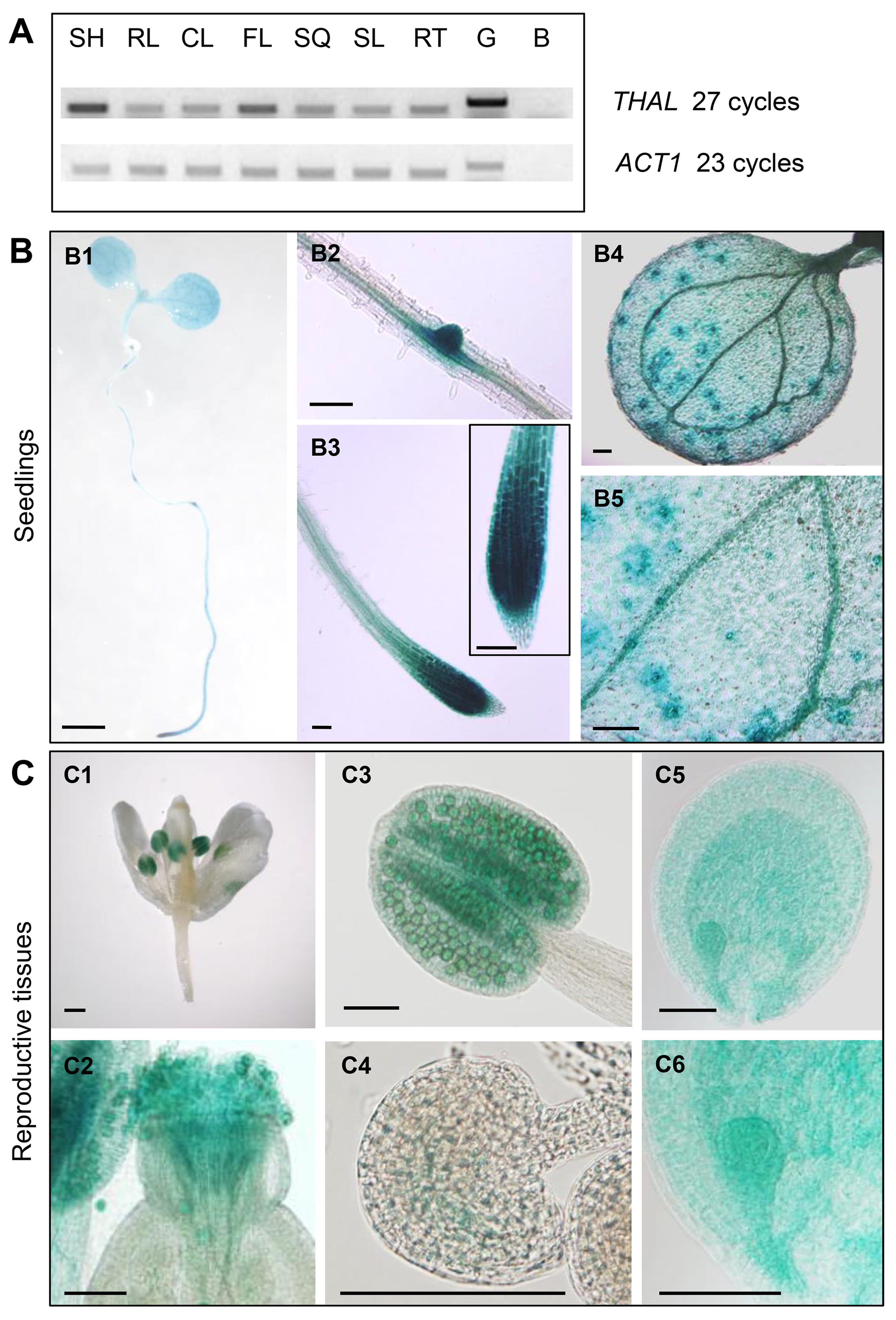

Supplement: S2 Fig — The expression profile of THAL in examined tissues by RT-PCR. ACT1 was an internal loading control to ensure equal loading of cDNA. Primer pair for ACT1 spanned an intron and revealed no genomic DNA contamination in all cDNAs. SH, shoot; RL, rosette leaf; CL, cauline leaf; FL, flower; SQ, silique; SL, seedling; RT, root; G, genomic DNA; B, blank without DNA template. THAL and ACT1 were PCR amplified with 27 and 23 cycles, respectively.(B–C) GUS staining of THALpro::GUS transgenic plants for THAL promoter activity in seedlings (B) and reproductive tissues (C). In seedlings (B1), GUS signals were significant in the lateral root primordia (B2), subapical region of the primary root (B3), leaf veins and around guard cells (B4 and B5). THALpro::GUS expression at the reproductive stage was observed in pollen and pollen tubes (C1-C3), ovule (C4), endosperm and embryo (C5 and C6). Scale bars = 0.2 mm (B1 and C1) and 100 μm (B2-B5, C2-C6). (TIF) [file pgen.1006408.s002.tif]

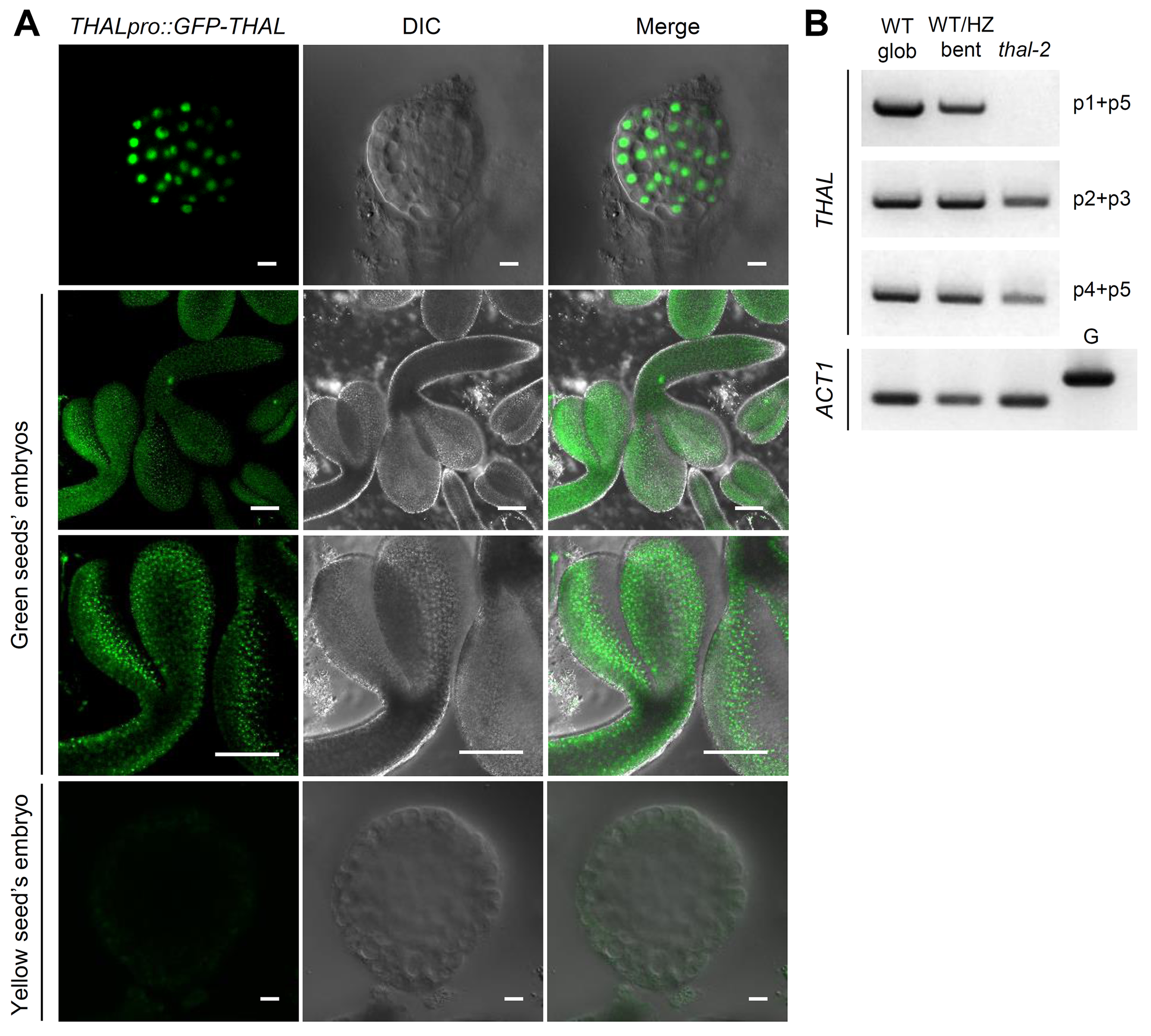

Supplement: S3 Fig — Embryo development in rescued thal-2 siliques. GFP signals in complemented globular and bent cotyledon embryos in green seeds and non-complemented globular embryos in yellow seeds (T2 seeds) from THALpro::GFP-THAL /thal-2 T1 plant. Scale bars = 10 μm (globular embryos) and 100 μm (bent cotyledon embryos).RT-PCR of globular WT (WT glob), bent cotyledon WT/HZ (WT/HZ bent), and thal-2 seeds using indicated primers (positions shown in Fig 1A). ACT1 was an internal control. G, genomic DNA. (TIF) [file pgen.1006408.s003.tif]

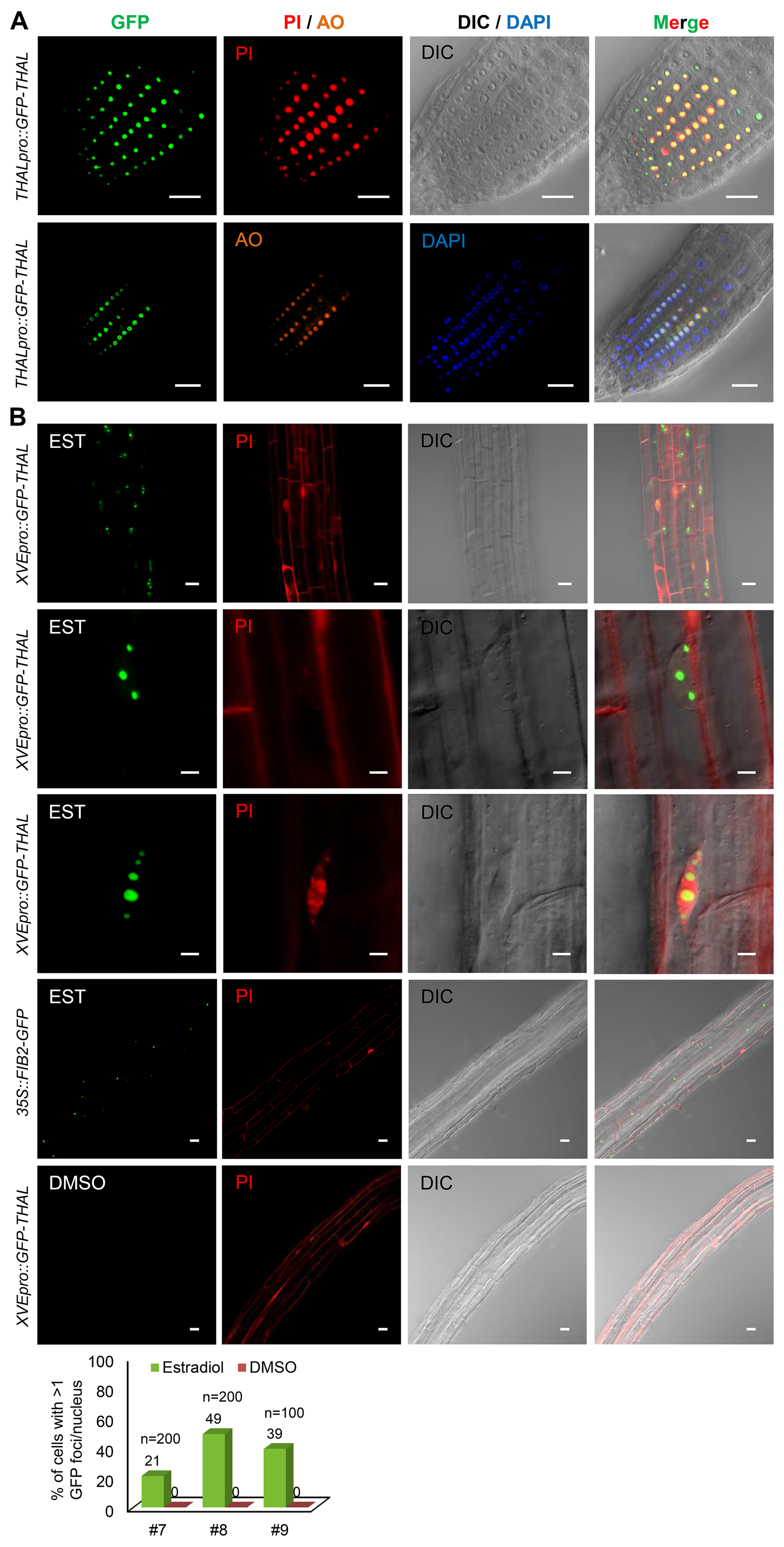

Supplement: S4 Fig — Confocal microscopy of roots of 10-d-old seedlings expressing THALpro::GFP-THAL (A) and 14-d-old seedlings expressing XVEpro::GFP-THAL (B). Propidium iodide (PI) or DAPI was used to stain nuclei. Acridine orange (AO) was used to mark nucleoli and only red fluorescence was registered.THAL localized in nucleoli of THALpro::GFP-THAL plants. Scale bars = 20 μm.THAL localized in multiple nucleoli within a PI-stained nucleus of XVEpro::GFP-THAL plants upon estradiol treatment. 35S::FIB2-GFP seedlings treated with estradiol did not display multiple nucleoli per nucleus. DMSO treatment did not induce GFP signals. Scale bars = 20 μm (images showing numerous cells) or 5 μm (single cell). Cells containing multiple GFP foci within a nucleus were quantified in three individual lines (Total cells n = 200, 200, 100 for #7, #8, #9, respectively). (TIF) [file pgen.1006408.s004.tif]

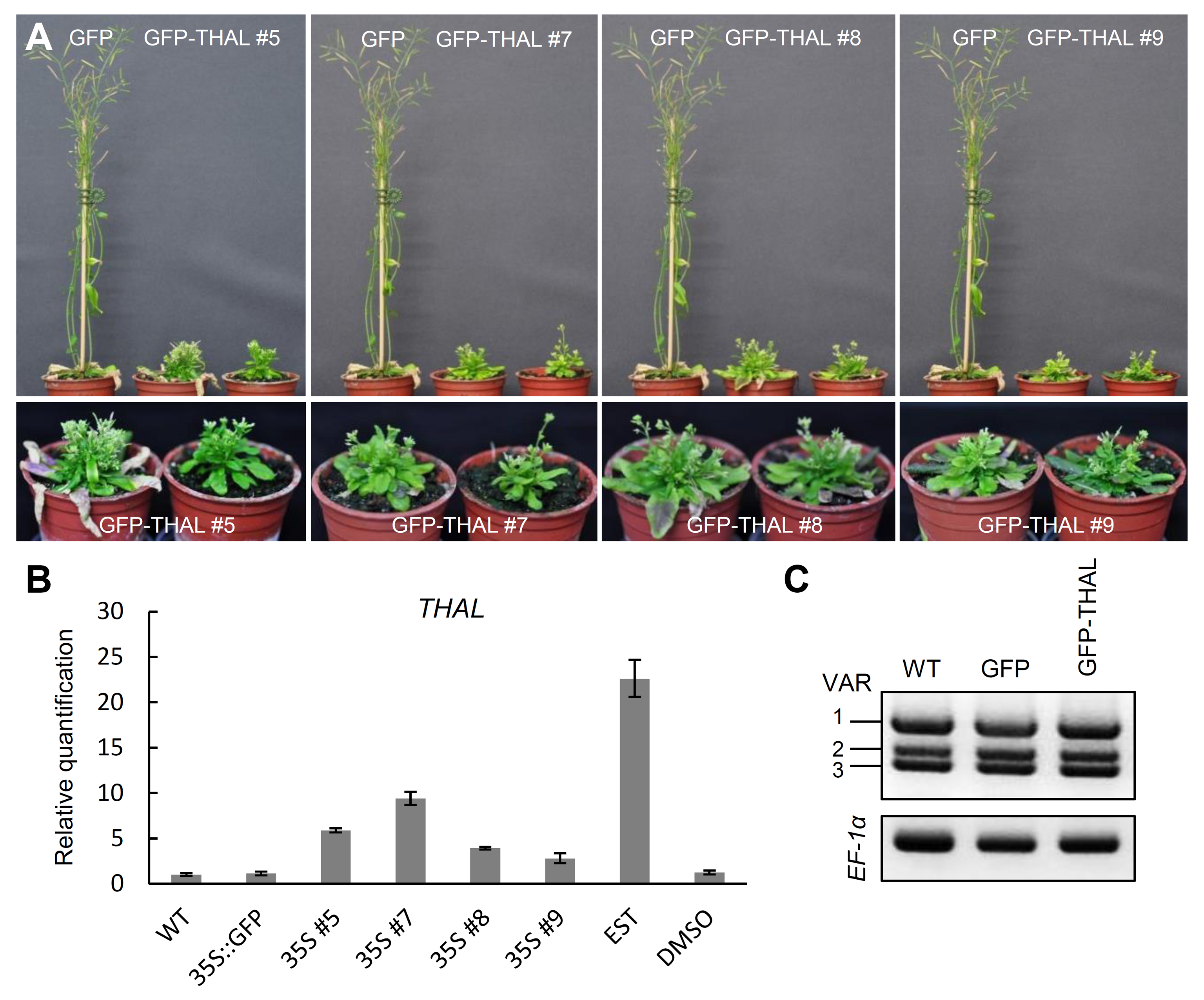

Supplement: S5 Fig — Phenotype of 35S::GFP-THAL plants, showing lines #5, 7, 8, and 9.Quantitative RT-PCR analysis of THAL expression in 35S::GFP-THAL (lines #5, 7, 8, and 9), 35S::GFP, and XVEpro::GFP-THAL upon estradiol (EST) or DMSO treatment relative to that in WT. Data are represented as means ± SD (n = 4).PCR analysis of rDNA variants using the primer pair shown in Fig 3C. EF-1α was an internal control. (TIF) [file pgen.1006408.s005.tif]

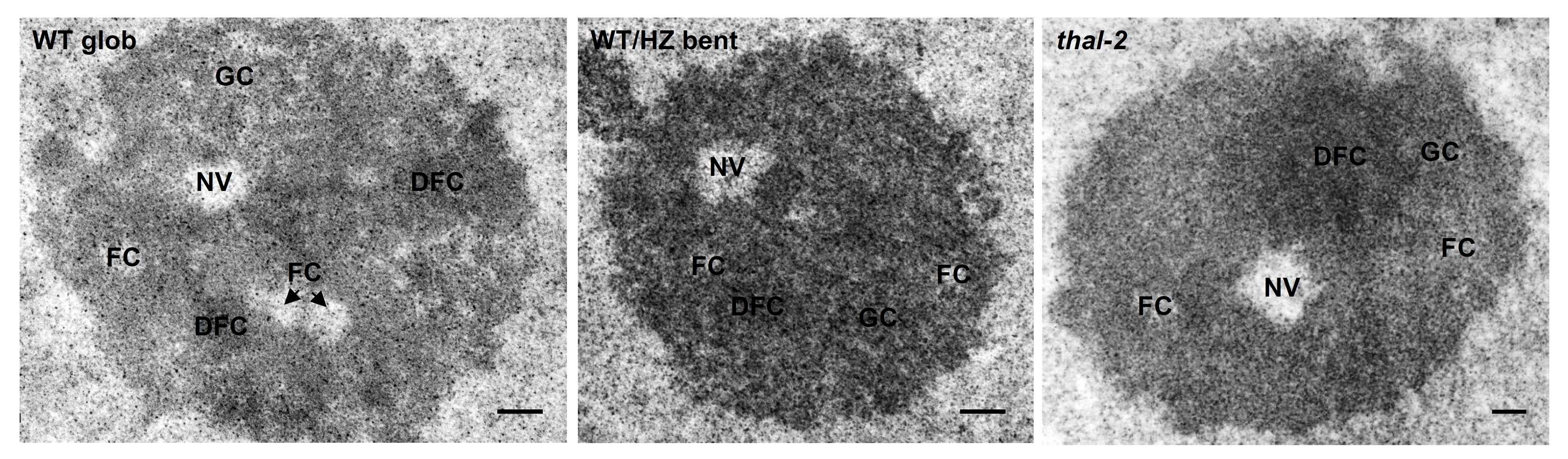

Supplement: S6 Fig — TEM images of subnucleolar structures in WT globular, WT/HZ bent cotyledon, and thal-2 embryos. FC, fibrillar center; DFC, dense fibrillar component; GC, granular component; NC, nucleolar vacuole. Scale bars = 200 μm. (TIF) [file pgen.1006408.s006.tif]

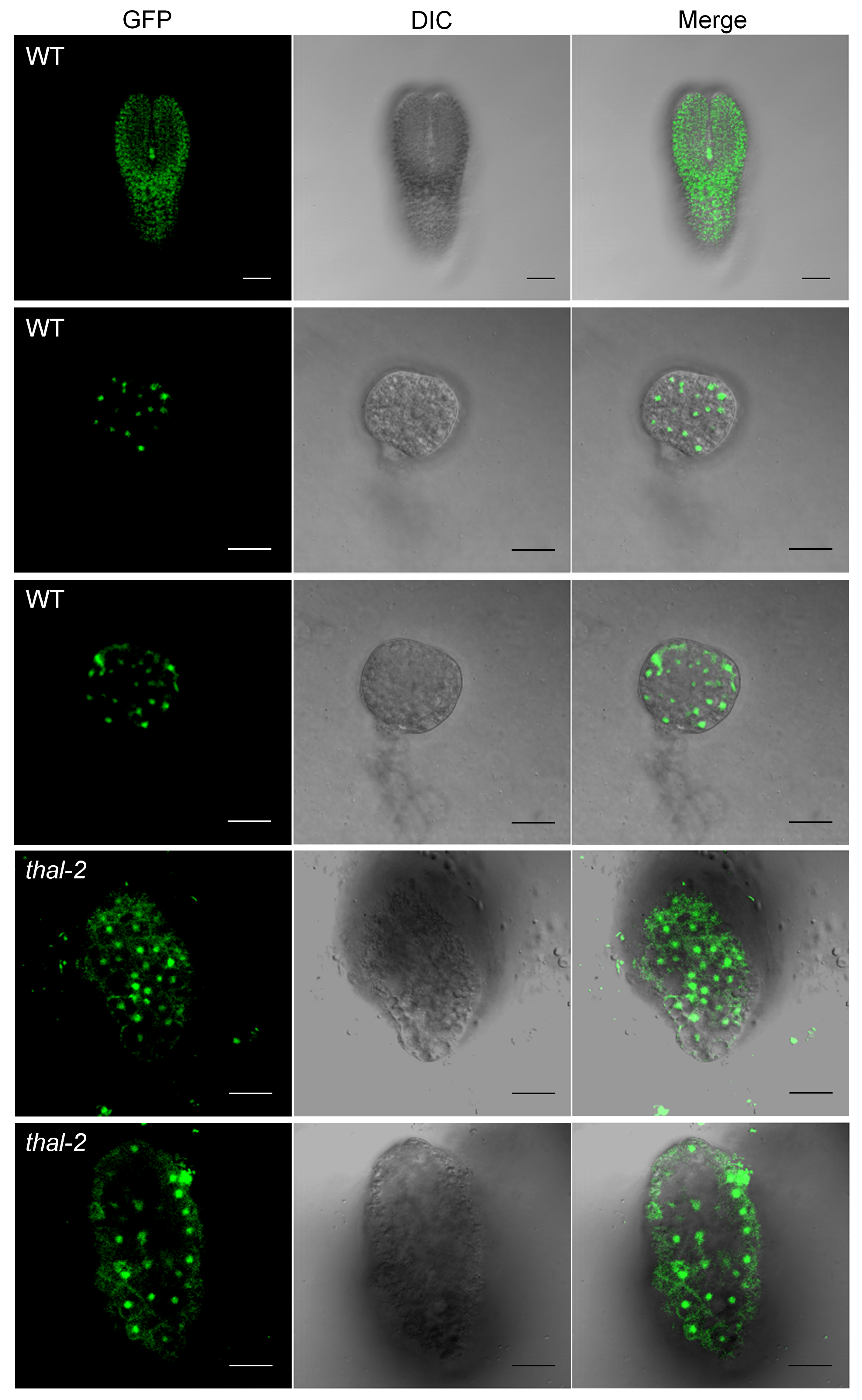

Supplement: S7 Fig — Nucleolar size of WT torpedo, WT globular, and thal-2 embryonic cells shown by nucleolar marker 35S::FIB2-GFP. Scale bars = 20 μm. WT globular and thal-2 embryo images are shown in the same scale. (TIF) [file pgen.1006408.s007.tif]

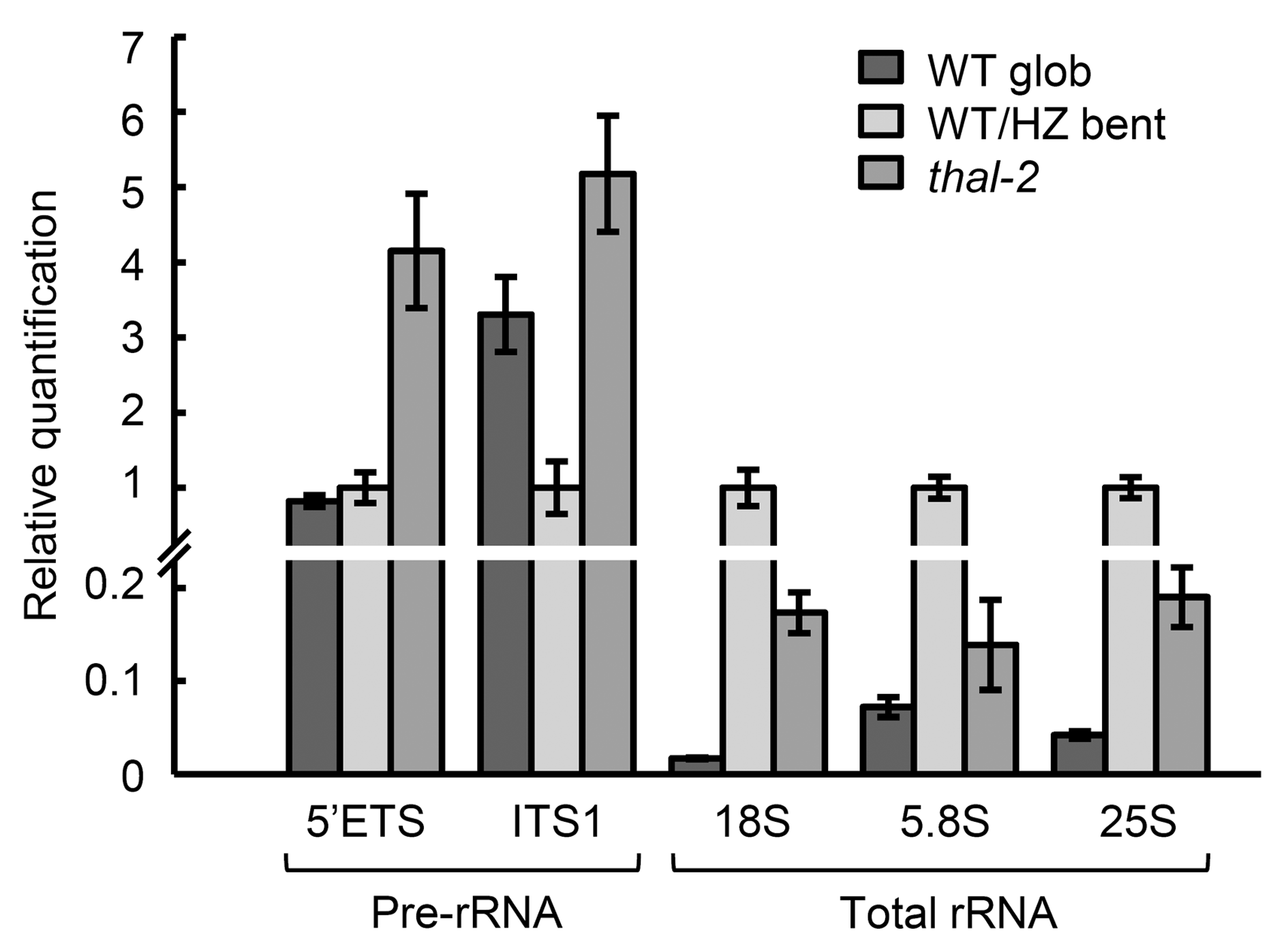

Supplement: S8 Fig — Quantitative RT-PCR of rRNA fragments normalized to ACT 1 in WT glob, WT/HZ bent, and thal-2 seeds (corresponding to Fig 4D). Data are represented as means ± SD (n = 10). (TIF) [file pgen.1006408.s008.tif]

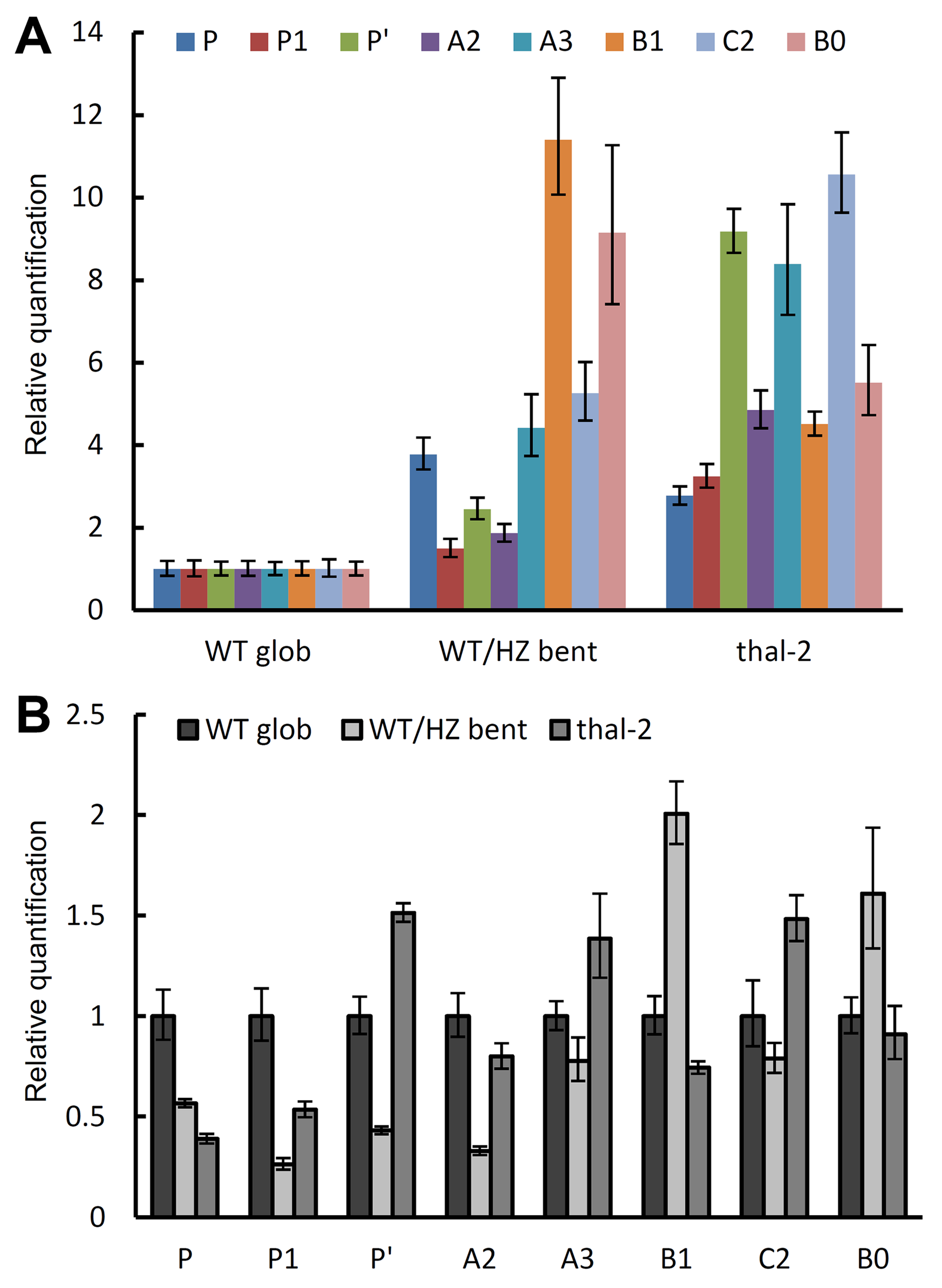

Supplement: S9 Fig — Quantitative RT-PCR of processing sites (normalized to EF-1α) in WT glob, WT/HZ bent, and thal-2 seeds, using WT glob to standardize (corresponding to Fig 5B). thal-2 seeds still showed an overall opposite amplification pattern as 35S::GFP-THAL (Fig 5B). Quantitative RT-PCR of processing sites (normalized to 45S precursors) in WT glob, WT/HZ bent, and thal-2 seeds, using WT glob to standardize (corresponding to Fig 5C). (TIF) [file pgen.1006408.s009.tif]

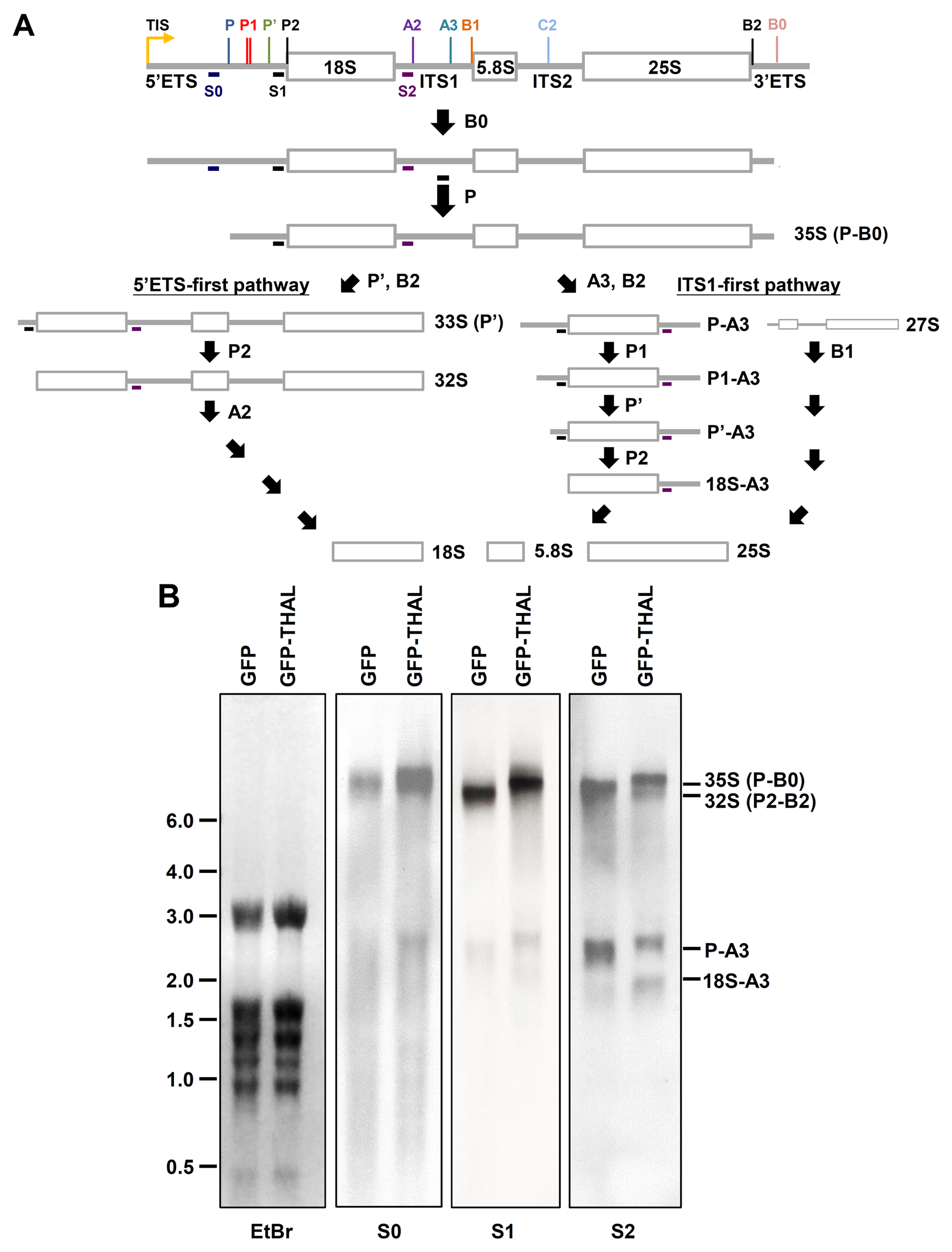

Supplement: S10 Fig — Schematic illustration of pre-rRNA processing events in Arabidopsis, showing processing site cleavages and precursors relevant to this study. Positions of S0, S1, and S2 probes used for northern blot analysis are indicated (blue, black, and purple bars, respectively). First cleavage at B0 site terminates transcription. The following 5’ splicing and P site cleavage generate 35S precursor, which can be processed by two alternative pathways to produce mature rRNAs. Northern blot analysis of processing in 35S::GFP-THAL. Ethidium bromide (EtBr) staining is shown as a loading control. S0 probe detected pre-rRNAs containing the fragment upstream of P site. Using the S1 probe, 35S and P-A3 precursors were detected in 35S::GFP (other intermediates are less detectable [18]). Fragments larger than 35S and P-A3 were detected in35S::GFP-THAL, suggesting attenuated cleavage at P site. S2 probe detected those recognized by S1 probe as well as 32S and 18S-A3 fragments. (TIF) [file pgen.1006408.s010.tif]

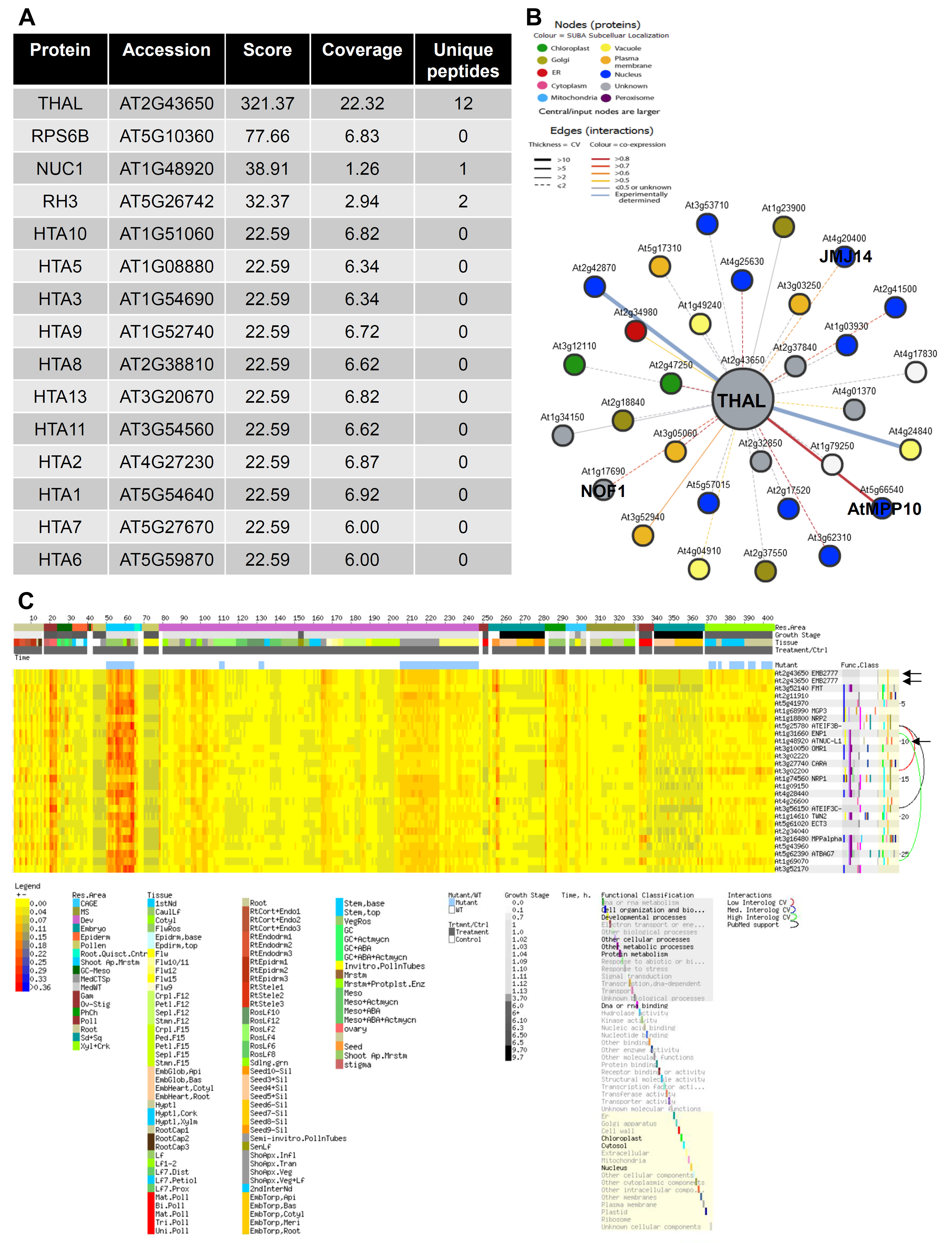

Supplement: S11 Fig — Graph shows interesting associated proteins of THAL detected by IP-MS using 8-d-old THALpro::GFP-THAL seedlings, eliminating those detected in WT. Putative interacting proteins of THAL presented by the Arabidopsis Interaction Viewer using the Bio-Analytic Resource (BAR) database. Co-expression analysis of THAL by the BAR Expression Angler. THAL (EMB2777) and NUC1 (ATNUC-L1) are indicated by black arrows on the right. (TIF) [file pgen.1006408.s011.tif]

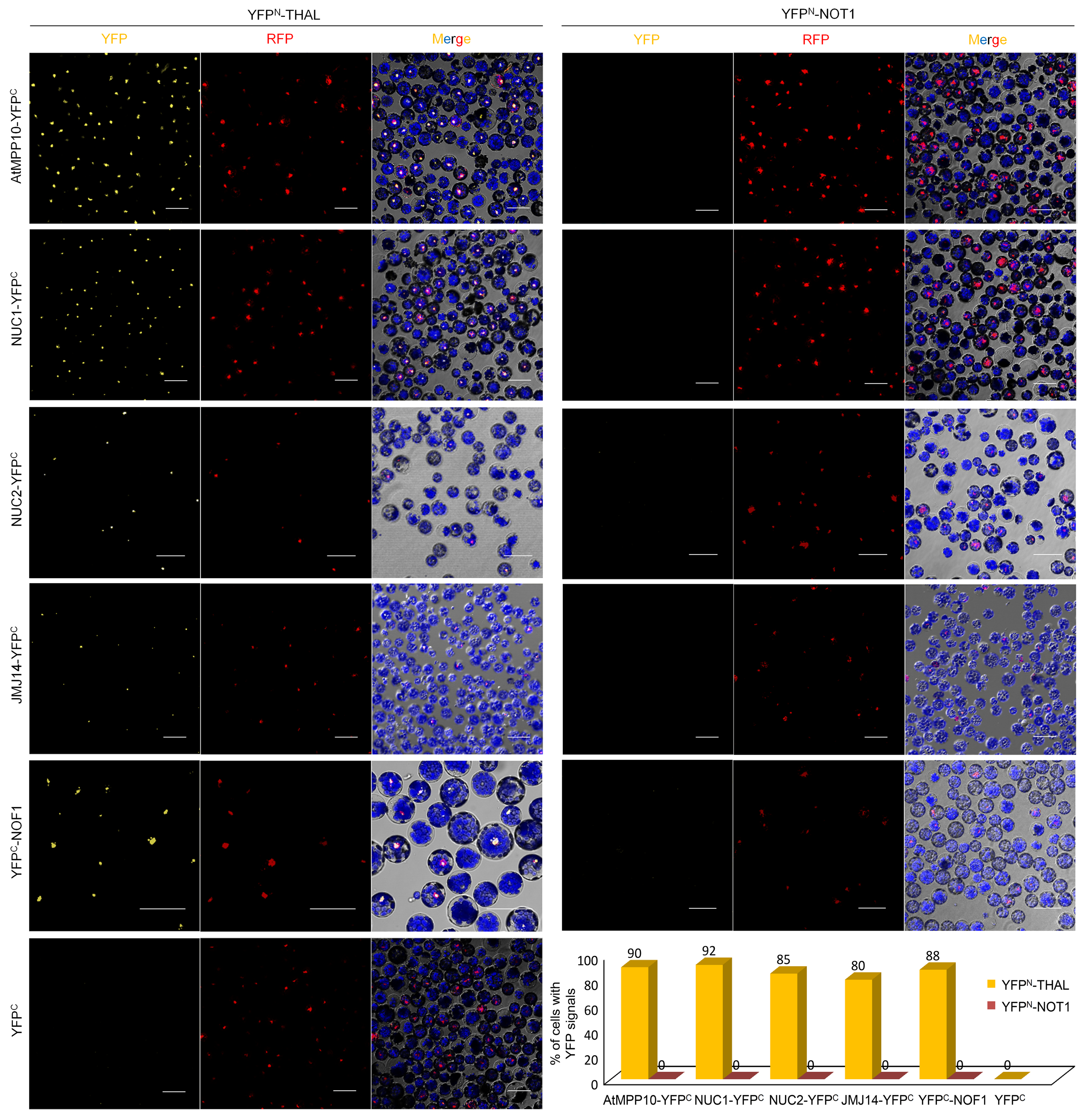

Supplement: S12 Fig — An unrelated protein YFPN-NOT1 and YFPC were used as negative controls. RFP fused to a nuclear localization signal was co-transformed as a marker for transformation efficiency and nuclei. Scale bars = 50μm. Total of 100 cells containing YFP signals were quantified for each interaction, and only cells with RFP signals were taken into account. (TIF) [file pgen.1006408.s012.tif]
