# Supplementary material for: Dual Role of a SAS10/C1D Family Protein in Ribosomal RNA Gene Expression and Processing Is Essential for Reproduction in Arabidopsis thaliana
Source: PLoS Genet. 2016 Oct 28;12(10):e1006408. doi: 10.1371/journal.pgen.1006408 (PMC5085252; doi:10.1371/journal.pgen.1006408)
Supplement: S1 Table — (PDF) [file pgen.1006408.s013.pdf]

S1 Table. Segregation ratio of germinated progeny of *thal-1/+*.

thal-1/+	**SQ1**	**SQ2**	**SQ3**	**SQ4**	**SQ5**	**SQ6**	**Total**	**%**
**HZ**	15	17	15	17	16	15	95	52.78
**WT**	15	13	15	14	12	16	85	47.22
**Total**	30	30	30	31	28	31	180	100

[a]$\chi 2$ (*P* value) = 0.46 (HZ:WT=1:1, *P* > 0.05, Student's *t* test).

[b]SQ: silique; HZ: heterozygote; WT: wild type.
