# Supplementary material for: Dual Role of a SAS10/C1D Family Protein in Ribosomal RNA Gene Expression and Processing Is Essential for Reproduction in Arabidopsis thaliana
Source: PLoS Genet. 2016 Oct 28;12(10):e1006408. doi: 10.1371/journal.pgen.1006408 (PMC5085252; doi:10.1371/journal.pgen.1006408)
Supplement: S2 Table — (PDF) [file pgen.1006408.s014.pdf]

S2 Table. Segregation ratio of progeny of *thal-2/+*.

thal-2/+	**SQ1**	**SQ2**	**SQ3**	**SQ4**	**SQ5**	**SQ6**	**Total**	**%**
**HM[a]**	12	9	11	10	9	8	59	22.605
**HZ**	25	26	21	16	27	30	145	55.556
**WT**	8	9	15	11	6	8	57	21.839
**Total**	45	44	47	37	42	46	261	100

[a]Ungerminated seeds.

[b]$\chi 2$ (*P* value) = 0.12 (HZ:WT=2:1, *P* > 0.05, Student's *t* test).

[c]SQ: silique; HM: homozygote; HZ: heterozygote; WT: wild type.
