# Supplementary material for: Dual Role of a SAS10/C1D Family Protein in Ribosomal RNA Gene Expression and Processing Is Essential for Reproduction in Arabidopsis thaliana
Source: PLoS Genet. 2016 Oct 28;12(10):e1006408. doi: 10.1371/journal.pgen.1006408 (PMC5085252; doi:10.1371/journal.pgen.1006408)
Supplement: S3 Table — (PDF) [file pgen.1006408.s015.pdf]

S3 Table. Primers used in this study.

Name	Sequence (5' to 3')	Purpose
SALK_016916 LP	GAAGTGTCGACATCTGCAACC	Genotyping of *thal-1*
SALK_016916 RP	CTTTTGCACTGCTCCATTAGC	Genotyping of *thal-1*
SALK_036872 LP	ACAAGAGCAAAACGAAGCATG	Genotyping of *thal-2*
SALK_036872 RP	CAGCTGATAAGCTCACCAAGC	Genotyping of *thal-2*
LBa1	TGGTTCACGTAGTGGGCCATCG	Genotyping of *thal* mutants
p1	ATGGGGAAAAAAGGAGGGACTTTG	RT-PCR of *THAL*
p2	GAAGATACAGAAGACGAAGAGGAGGC	RT-PCR of *THAL*
p3	TCTTCACTATCATCGTCTAACCCAG	RT-PCR of *THAL*
p4	GAGATGATATTGGAGAACGGCGTAG	RT-PCR of *THAL*
p5	CTACATTCGGATGCTCCGGCTTG	RT-PCR of *THAL*
THAL cDNA-F (SalI)	ACGCAGTCGACCATGGGGAAAAAAGGAGGGACTTTG	Protoplast transient expression construct
THAL cDNA-R (SalI)	ACGCAGTCGACCATTCGGATGCTCCGGCTTG	Protoplast transient expression construct
THALΔC-R0 (SalI)	ACGCAGTCGACGGGAAGCTCTTCATCAAGTTCTT	Protoplast transient expression construct
THALΔC-R1 (SalI)	ACGCAGTCGACTTCTGGCGATGACGGCATCA	Protoplast transient expression construct
THALΔC-R2 (SalI)	ACGCAGTCGACTCTGACCTGTCCTTTGCGTCTAG	Protoplast transient expression construct
THALΔN-F (SalI)	ACGCAGTCGACCATGGGGGATCAAAGAACTTGATGA AGAGC	Protoplast transient expression construct
FIB2 CDS-F (SalI)	ACGCAGTCGACCATGAGACCTCCTCTAACTGGAAG	Protoplast transient expression construct
FIB2 CDS-R (SalI)	ACGCAGTCGACCTAAGCAGCAGTAGCAGCCT	Protoplast transient expression construct
THAL full-F (XmaI)	CCCCCCGGGCTTTCTTCTCTGCCTCTGTCGATTTCTTC	*THAL* complementation construct
THAL full-R (PstI)	AACTGCAGCTAAGCCTGGCACGCCGATC	*THAL* complementation construct
THAL gene-R (PstI)	AACTGCAGCATTCGGATGCTCCGGCTTG	*THAL* complementation construct
THALcDNA-R(XmaI)	CCCCCCGGGCATTCGGATGCTCCGGCTTG	*THAL* complementation construct
35S-F (XmaI)	CCCCCCGGGCAGTGCCTCGCTACCTTAG	*THAL* complementation construct
GFP-F (PstI)	AACTGCAGATGGTGAGCAAGGGCGAGG	*THAL* complementation construct
THAL out-R	AGGGCCAAGGGAGTTGGTGTGT	Genotyping for *THAL* complementation
THAL pro-R (XmaI)	CCCCCCGGGTGTTGTGTTGCAGAGCAGGTTCTG	GUS analysis of *THAL* promoter activity
rDNA 5'ETS-F	CCTTGCTCGCATTGGTGAAT	qRT-PCR for rRNA processing analysis
rDNA 5'ETS-R	CGTCGACAACTTTTCCGCAT	qRT-PCR for rRNA processing analysis
rDNA 18S-F	TGACGGAGAATTAGGGTTC	qRT-PCR for rRNA processing analysis
rDNA 18S-R	CCTCCAATGGATCCTCGTTA	qRT-PCR for rRNA processing analysis
rDNA ITS1-F	CGCGAACCAAAGATCACCA	qRT-PCR for rRNA processing analysis
rDNA ITS1-R	GGCAAGGAATCGGCTAAGAAA	qRT-PCR for rRNA processing analysis
rDNA 5.8S-F	GCAACGGATATCTCGGCTCTC	qRT-PCR for rRNA processing analysis
rDNA 5.8S-R	TGCGTTCAAAGACTCGATGG	qRT-PCR for rRNA processing analysis
rDNA 25S-F	CTACCGTGCGCTGGATTATGA	qRT-PCR for rRNA processing analysis
rDNA 25S-R	GCTTCTAGCCCGGATTCTGACT	qRT-PCR for rRNA processing analysis
rDNA P-F	CCGCCTAGGCTGTCCCGAAG	qRT-PCR for P site cleavage analysis
rDNA P-R	CGGACGAATCCAAGCCAAAG	qRT-PCR for P site cleavage analysis
rDNA P1-F	CTTTGGCTTGGATTCGTCCG	qRT-PCR for P1 site cleavage analysis
rDNA P1-R	CGTGAATTAACTGAGAAGGA	qRT-PCR for P1 site cleavage analysis
rDNA P'-F	GTTGTCGACGGTGACTCGAAGT	qRT-PCR for P' site cleavage analysis
rDNA P'-R	GGTCTTGACCGTCATCTTTTGC	qRT-PCR for P' site cleavage analysis

rDNA A2-F	TGAACCTGCGGAAGGATCAT	qRT-PCR for A2 site cleavage analysis
rDNA A2-R	CGCGAACCAAAGATCACCA	qRT-PCR for A2 site cleavage analysis
rDNA A3-F	AAAACCCCAGCACGAAAAGTGT	qRT-PCR for A3 site cleavage analysis
rDNA A3-R	AATGCCAGCCGTTCGTTTG	qRT-PCR for A3 site cleavage analysis
rDNA B1-F	ATGCTGTGCTGCGAACTGAA	qRT-PCR for B1 site cleavage analysis
rDNA B1-R	GAGATATCCGTTGCCGAGAGTC	qRT-PCR for B1 site cleavage analysis
rDNA C2-F	TAAATCCGAGCCAAGGACG	qRT-PCR for C2 site cleavage analysis
rDNA C2-R	TCTACTGCTTCCGGACAAGAGC	qRT-PCR for C2 site cleavage analysis
rDNA B0-F	AACAATCCCCAATTCTACACAAGTG	qRT-PCR for B0 site cleavage analysis
rDNA B0-R	ACCTCGAGAGACGAGCTCGTGTA	qRT-PCR for B0 site cleavage analysis
45S-F	GGGGGGTGGGTGTTGAGGGAG	qRT-PCR for 45S pre-rRNA
45S-R	GAAAAAGGGGGTTCCCACGGAC	qRT-PCR for 45S pre-rRNA
VAR-F	GACAGACTTGTCCAAAACGCCCACC	RT-PCR of rDNA variants
VAR-R	CTGGTCGAGGAATCCTGGACGATT	RT-PCR of rDNA variants
5’EF-1α	CTAAGGATGGTCAGACCCG	Internal control
3’EF-1α	CTTCAGGTATGAAGACACC	Internal control
ACT1-F	CATCAGGAAGGACTTGTACGG	Internal control
ACT1-R	GATGGACCTGACTCGTCATAC	Internal control
rDNA 5’ETS-F	CCTTGCTCGCATTGGTGAAT	qRT-PCR for rRNA processing analysis
rDNA 5’ETS-R	CGTCGACAACTTTTCCGCAT	qRT-PCR for rRNA processing analysis
rDNA 18S-F	TGACGGAGAATTAGGGTTC	qRT-PCR for rRNA processing analysis
rDNA 18S-R	CCTCCAATGGATCCTCGTTA	qRT-PCR for rRNA processing analysis
rDNA ITS1-F	CGCGAACCAAAGATCACCA	qRT-PCR for rRNA processing analysis
rDNA ITS1-R	GGCAAGGAATCGGCTAAGAAA	qRT-PCR for rRNA processing analysis
rDNA 5.8S-F	GCAACGGATATCTCGGCTCTC	qRT-PCR for rRNA processing analysis
rDNA 5.8S-R	TGCGTTCAAAGACTCGATGG	qRT-PCR for rRNA processing analysis
rDNA 25S-F	CTACCGTGCGCTGGATTATGA	qRT-PCR for rRNA processing analysis
rDNA 25S-R	GCTTCTAGCCCGGATTCTGACT	qRT-PCR for rRNA processing analysis
rDNA P-F	CCGCCTAGGCTGTCCCGAAG	qRT-PCR for P site cleavage analysis
rDNA P-R	CGGACGAATCCAAGCCAAAG	qRT-PCR for P site cleavage analysis
rDNA P1-F	CTTTGGCTTGGATTCGTCCG	qRT-PCR for P1 site cleavage analysis
rDNA P1-R	CGTGAATTAACTGAGAAGGA	qRT-PCR for P1 site cleavage analysis
rDNA P’-F	GTTGTCGACGGTGACTCGAAGT	qRT-PCR for P’ site cleavage analysis
rDNA P’-R	GGTCTTGACCGTCATCTTTTGC	qRT-PCR for P’ site cleavage analysis
rDNA A2-F	TGAACCTGCGGAAGGATCAT	qRT-PCR for A2 site cleavage analysis
rDNA A2-R	CGCGAACCAAAGATCACCA	qRT-PCR for A2 site cleavage analysis
rDNA A3-F	AAAACCCCAGCACGAAAAGTGT	qRT-PCR for A3 site cleavage analysis
rDNA A3-R	AATGCCAGCCGTTCGTTTG	qRT-PCR for A3 site cleavage analysis
rDNA B1-F	ATGCTGTGCTGCGAACTGAA	qRT-PCR for B1 site cleavage analysis
rDNA B1-R	GAGATATCCGTTGCCGAGAGTC	qRT-PCR for B1 site cleavage analysis
rDNA C2-F	TAAATCCGAGCCAAGGACG	qRT-PCR for C2 site cleavage analysis
rDNA C2-R	TCTACTGCTTCCGGACAAGAGC	qRT-PCR for C2 site cleavage analysis
rDNA B0-F	AACAATCCCCAATTCTACACAAGTG	qRT-PCR for B0 site cleavage analysis
rDNA B0-F	ACCTCGAGAGACGAGCTCGTGTA	qRT-PCR for B0 site cleavage analysis
45S-F	GGGGGGTGGGTGTTGAGGGAG	qRT-PCR for 45S pre-rRNA

45S-R	GAAAAAGGGGGTTCCCACGGAC	qRT-PCR for 45S pre-rRNA
VAR-F	GACAGACTTGTCCAAAACGCCCACC	RT-PCR of rDNA variants
VAR-R	CTGGTCGAGGAATCCTGGACGATT	RT-PCR of rDNA variants
5'EF-1α	CTAAGGATGGTCAGACCCG	Internal control
3'EF-1α	CTTCAGGTATGAAGACACC	Internal control
ACT1-F	CATCAGGAAGGACTTGTACGG	Internal control
ACT1-R	GATGGACCTGACTCGTCATAC	Internal control

[a]The underlined sequences denote restriction enzyme sites.
