# Supplementary material for: Dual Role of a SAS10/C1D Family Protein in Ribosomal RNA Gene Expression and Processing Is Essential for Reproduction in Arabidopsis thaliana
Source: PLoS Genet. 2016 Oct 28;12(10):e1006408. doi: 10.1371/journal.pgen.1006408 (PMC5085252; doi:10.1371/journal.pgen.1006408)
Supplement: S1 Text — (PDF) [file pgen.1006408.s016.pdf]

S1 Text. Supporting materials and methods.

THAL Expression Analysis by RT-PCR and GUS Activity Staining

Total RNA was extracted from various plant tissues using the Plant Total RNA Miniprep Purification Kit (GeneMark) and the TURBO DNA-free Kit (Applied Biosystems) to remove DNA contamination. *THAL* and internal control *ACT1* were amplified by use of primer pairs shown in S3 Table. To observe GUS expression patterns, *THALpro::GUS* transgenic plants from at least 5 individual lines were stained with GUS staining solution for 5 hr, then transferred to 70% ethanol to remove chlorophyll before observation.

Fluorescence Staining

To observe the localization of THAL in transgenic plants, 10 µM propidium iodide (PI; Sigma) or 0.2 µg/ml DAPI (Sigma) were used to stain nuclei and 5 µg/ml acridine orange (AO; Invitrogen) in 0.03 M phosphate buffer, pH 6.8 was used for nucleolar staining.

Estradiol Induction of XVE Expression System

XVEpro::GFP-THAL and negative control *35S::FIB2-GFP* 8-d-old seedlings were transferred to half MS plates containing 8 µM β-estradiol or DMSO for 5 d before confocal observation.

Northern Blot Analysis

Total RNA was isolated from 5-wk-old *35S::GFP-THAL* and *35S::GFP* leaves using RNeasy Plant Mini Kit (Qiagen) and treated with TURBO DNA-free Kit (Applied Biosystems). Six micrograms of total RNA was separated on a 1.2% agarose/formaldehyde gel in MOPS buffer and transferred to a Hybond N+ membrane (GE Healthcare) by capillary method. RNA was fixed to the membrane by incubating at 80℃ for 2 hr. Hybridization was performed overnight at 55℃ for S0 probe (5’-GGACAGCCTAGGCGGATCCATGCTTTCCAAC-3’) and S1 probe (5’-ACGGCAATTCCCCGCCACATCCTCTCAAAC-3’) and at 45℃ for S2 probe (5’-GTCGTTCTGTTTTGGACAGGTATCGA-3’). After stringent washes, signals were detected by chemiluminescent method.

Immunoprecipitation and MS Analysis

Total proteins were extracted from 5 g of 8-d-old *THALpro::GFP-THAL/thal-2* and WT seedlings. THAL associated proteins were co-immunoprecipitated using GFP-trap beads (Chromotek) according to the manufacturer’s manual, except that

washing was done with extraction buffer.
For MS analysis, the LC-nESI-Q Exactive mass spectrometer model (Thermo Fisher Scientific) coupled with an online nanoUHPLC (Dionex UltiMate 3000 Binary RSLCnano) was used. Peptide and protein identification was performed using the Proteome Discoverer software (v1.4, Thermo Fisher Scientific) with Mascot (v. 2.4) search engine against a TAIR10 database with 27,416 protein sequence entries.
